# Endothelial TRPV4 channels modulate vascular tone by Ca^2+^‐induced Ca^2+^ release at inositol 1,4,5‐trisphosphate receptors

**DOI:** 10.1111/bph.14762

**Published:** 2019-07-24

**Authors:** Helen R. Heathcote, Matthew D. Lee, Xun Zhang, Christopher D. Saunter, Calum Wilson, John G. McCarron

**Affiliations:** ^1^ Strathclyde Institute of Pharmacy and Biomedical Science University of Strathclyde Glasgow UK; ^2^ Centre for Advanced Instrumentation, Biophysical Sciences Institute, Department of Physics Durham University Durham UK

## Abstract

**Background and Purpose:**

The TRPV4 ion channels are Ca^2+^ permeable, non‐selective cation channels that mediate large, but highly localized, Ca^2+^ signals in the endothelium. The mechanisms that permit highly localized Ca^2+^ changes to evoke cell‐wide activity are incompletely understood. Here, we tested the hypothesis that TRPV4‐mediated Ca^2+^ influx activates Ca^2+^ release from internal Ca^2+^ stores to generate widespread effects.

**Experimental Approach:**

Ca^2+^ signals in large numbers (~100) of endothelial cells in intact arteries were imaged and analysed separately.

**Key Results:**

Responses to the TRPV4 channel agonist GSK1016790A were heterogeneous across the endothelium. In activated cells, Ca^2+^ responses comprised localized Ca^2+^ changes leading to slow, persistent, global increases in Ca^2+^ followed by large propagating Ca^2+^ waves that moved within and between cells. To examine the mechanisms underlying each component, we developed methods to separate slow persistent Ca^2+^ rise from the propagating Ca^2+^ waves in each cell. TRPV4‐mediated Ca^2+^ entry was required for the slow persistent global rise and propagating Ca^2+^ signals. The propagating waves were inhibited by depleting internal Ca^2+^ stores, inhibiting PLC or blocking IP_3_ receptors. Ca^2+^ release from stores was tightly controlled by TRPV4‐mediated Ca^2+^ influx and ceased when influx was terminated. Furthermore, Ca^2+^ release from internal stores was essential for TRPV4‐mediated control of vascular tone.

**Conclusions and Implications:**

Ca^2+^ influx via TRPV4 channels is amplified by Ca^2+^‐induced Ca^2+^ release acting at IP_3_ receptors to generate propagating Ca^2+^ waves and provide a large‐scale endothelial communication system. TRPV4‐mediated control of vascular tone requires Ca^2+^ release from the internal store.

**Abbreviations**
2‐APB: 2‐aminoethoxydiphenyl borate
CPA: cyclopiazonic acid
F: fluorescence intensity
F_0_: baseline fluorescence intensity
GSK: GSK1016790A
HC067: HC067047
IP_3_: inositol 1,4,5‐trisphosphate
IP_3_R: inositol 1,4,5‐trisphosphate receptor
RuR: ruthenium red
RyRs: ryanodine receptors
SNP: sodium nitroprusside

**What is already known**

- TRPV4 channels are key Ca^2+^ permeable channels that control endothelial function.
- TRPV4 channels generate large but highly localized Ca^2+^ signals.

**What this study adds**

- Ca^2+^‐induced Ca^2+^ release from IP_3_‐sensitive internal store amplifies local Ca^2+^ signals from TRPV4 channel activity.
- Ca^2+^‐induced Ca^2+^ release at IP_3_ receptors mediates control of vascular contractility by endothelial TRPV4 channels.

**What is the clinical significance**

- Endothelial TRPV4 channels are involved in several disease conditions such as cancer progression and hypertension.
- Endothelial TRPV4‐mediated Ca^2+^‐induced Ca^2+^ release at IP_3_ receptors offers a new target for drug development.

## 1 INTRODUCTION

The endothelium plays a critical role in the regulation of numerous vascular processes such as maintaining vascular tone, regulating the passage of macromolecules and oxygen to tissues, modulating immune responses, initiating angiogenesis, and controlling vascular remodelling. The control by the endothelium of each process is mediated by the generation of various signalling molecules that include NO, prostacyclin, von Willebrand factor, tissue plasminogen activator, and endothelial‐derived hyperpolarizing and contracting factors (Taylor et al., 2003; Whorton, Willis, Kent, & Young, 1984). The generation of each of these signalling molecules is controlled tightly by changes in the cytoplasmic Ca^2+^ concentration in the endothelium. Central therefore to an understanding of endothelial function is an appreciation of the control of intracellular Ca^2+^ concentrations.

There are two major sources of Ca^2+^ for the endothelium: the extracellular space and the intracellular Ca^2+^ store. Ca^2+^ influx from the extracellular space is mediated mainly by channels present on the plasmalemmal membrane. In many cell types, such as smooth muscle and cardiomyocytes, the influx pathways are well characterized, and voltage‐dependent Ca^2+^ channels are major contributors (Catterall, 2011; Nelson, Patlak, Worley, & Standen, 1990). In endothelial cells, however, understanding of the Ca^2+^ influx pathways is incomplete (Nilius, Droogmans, & Wondergem, 2003). Several members of the transient receptor potential (TRP) superfamily of cation channels are present in endothelial cells (Hofmann, Schaefer, Schultz, & Gudermann, 2002; Nilius, Droogmans, & Wondergem, 2003). Of these, the TRPV4 channel has attracted substantial interest because of the channels' relatively high Ca^2+^ permeability (Filosa, Yao, & Rath, 2013; Watanabe et al., 2002). TRPV4 channels are expressed widely in endothelial cells (Mendoza et al., 2010; Sullivan, Francis, Pitts, Taylor, & Earley, 2012; Watanabe et al., 2002). TRPV4 channels were initially demonstrated to be activated by hypotonicity‐induced cell swelling (Liedtke et al., 2000; Mizuno, Matsumoto, Imai, & Suzuki, 2003). Later studies revealed that the channel is also activated by various other stimuli including mechanical stimulation (Gao, Wu, & O'Neil, 2003; Kohler et al., 2006; Liedtke, Tobin, Bargmann, & Friedman, 2003), moderate heat, endogenous chemicals such as anandamide, arachidonic acid, and its epoxyeicosatrienoic acid metabolites, as well as by a number of exogenous chemical ligands (Nilius & Voets, 2013; Vriens et al., 2004; Watanabe et al., 2003) or UVB (Moore et al., 2013; Yin et al., 2008). In the endothelium, by regulating the cytoplasmic Ca^2+^ concentration, TRPV4 channels may contribute to the response to arachidonic acid metabolites, physical forces, and agonists (Bagher et al., 2012; Freichel et al., 2001; Hartmannsgruber et al., 2007; Kohler et al., 2006; Marrelli, O'Neil, Brown, & Bryan, 2007; Mendoza et al., 2010; Watanabe et al., 2002; Zhang et al., 2009;Zhang, Papadopoulos, & Hamel, 2013).

While large in amplitude, the Ca^2+^ rise evoked by activation of endothelial TRPV4 channels is reported to be highly localized and confined to within a few micrometres of the channels (Sonkusare et al., 2012; Sonkusare et al., 2014). These highly localized Ca^2+^ signals evoke endothelial and smooth muscle hyperpolarization and vascular relaxation (F. Gao & Wang, 2010; Kohler et al., 2006; Ma et al., 2013; Sonkusare et al., 2012; D. X. Zhang et al., 2009). The highly localized nature of the Ca^2+^ signal provides tight spatial control of the cellular effectors activated, for example, Ca^2+^‐activated K^+^ channels (Gao & Wang, 2010; Ma et al., 2013; Sonkusare et al., 2012). However, more widespread Ca^2+^ rises throughout the cytoplasm have been implicated in the regulation of endothelial structure, maintenance of the normal orientation of endothelial cells, the control and selectively of endothelial permeability, and the production of antithrombotic factors. TRPV4 channels have been proposed to play a role in each of these processes (Noren et al., 2016; Phuong et al., 2017; Thodeti et al., 2009; Thoppil et al., 2016). The question arises as to how activation of TRPV4 channels may lead to Ca^2+^‐dependent events throughout the cytoplasm if the increase in Ca^2+^ concentration arising from TRPV4 channel activity remains localized to within a few microns of the channel.

The second major source of Ca^2+^ in endothelial cells is the internal Ca^2+^ store. Ca^2+^ release from the internal store may occur via ryanodine receptors (RyRs) or IP_3_ receptors (IP_3_Rs). While RyRs may be expressed in endothelial cells (Moccia, Berra‐Romani, & Tanzi, 2012; Mumtaz, Burdyga, Borisova, Wray, & Burdyga, 2011; Rusko, Wang, & Vanbreemen, 1995), the functional role of the channels in the endothelium is not clear given that RyR activators such as caffeine do not increase cytoplasmic Ca^2+^ in endothelial cells (Borisova, Wray, Eisner, & Burdyga, 2009; Wilson et al., 2019; Wilson, Lee, & McCarron, 2016). Ca^2+^ release from the internal store in endothelial cells may be mediated mainly by IP_3_Rs (Mumtaz et al., 2011; Wilson et al., 2019). Ca^2+^ release via IP_3_R occurs in response to physical forces and circulating vasoactive substances and contributes to the control of several vascular activities such as vasorelaxation, endothelial permeability, and production of anti‐thrombotic factors (Sun, Geyer, & Komarova, 2017).

IP_3_Rs are themselves regulated by Ca^2+^, in addition to IP_3_. Ca^2+^ released via IP_3_Rs may induce a positive feedback Ca^2+^‐induced Ca^2+^ release process at neighbouring IP_3_Rs. Indeed, Ca^2+^ signals at IP_3_Rs begin as a localized Ca^2+^ blip at a single receptor (∼1 μm spread) which expand by Ca^2+^‐induced Ca^2+^ release opening neighbouring IP_3_Rs generating a Ca^2+^ puff (∼4 μm spread). Puffs may further propagate via Ca^2+^‐induced Ca^2+^ release to form a transient global Ca^2+^ elevation throughout the cell or a Ca^2+^ “wave” (Berridge, 1997; Bootman, Niggli, Berridge, & Lipp, 1997). These waves may move through all or part of the cell (Bootman, Berridge, & Lipp, 1997). In some conditions that are not fully understood, IP_3_‐evoked Ca^2+^ waves may also move between cells to provide a signalling system capable of coordinating the activity of many cells (Leybaert & Sanderson, 2012).

While Ca^2+^ influx and release are activated separately, they are not independent: Ca^2+^ influx can trigger release, and release can trigger influx (McCarron, Chalmers, Bradley, Macmillan, & Muir, 2006). In cardiomyocytes, Ca^2+^ influx via voltage‐dependent Ca^2+^ channels may activate RyRs to evoke a large rise in Ca^2+^, inducing cell contraction. In smooth muscle cells, TRPV4 channel activity may lead to Ca^2+^‐induced Ca^2+^ release acting at RyRs (Earley, Heppner, Nelson, & Brayden, 2005). In astrocytes, Ca^2+^‐induced Ca^2+^ release occurs in response to Ca^2+^ influx via TRPV4 channels but at IP_3_Rs rather than at RyRs. This process amplifies and propagates the Ca^2+^ signal arising from TRPV4‐mediated Ca^2+^ influx (Dunn, Hill‐Eubanks, Liedtke, & Nelson, 2013). TRPV4‐mediated Ca^2+^ influx may also lead to recruitment of IP_3_Rs and Ca^2+^‐induced Ca^2+^ release in murine‐derived cultured neuronal cells (Shen et al., 2018). These observations raise the possibility of TRPV4‐mediated Ca^2+^ influx being able to generate a more global increase in intracellular Ca^2+^ via Ca^2+^‐induced Ca^2+^ release in endothelial cells.

To explore this possibility, we examined the role that the internal store plays in regulating alterations in intracellular Ca^2+^ evoked by activation of TRPV4 channels. Here, we report that the IP_3_‐sensitive Ca^2+^ store is critical for the TRPV4‐evoked global Ca^2+^ rise in endothelial cells in intact blood vessels. Ca^2+^ influx generates Ca^2+^‐induced Ca^2+^ release at IP_3_Rs to evoke Ca^2+^ waves in the vascular endothelium. IP_3_R‐dependent Ca^2+^ waves are required for the endothelium‐dependent vascular smooth muscle cell relaxation in response to activation of TRPV4 channels.

## 2 METHODS

### 2.1 Animals

All animal care and experimental procedures complied with the relevant UK Home Office Regulations, (Schedule 1 of the Animals [Scientific Procedures] Act 1986, UK) and were approved by the University of Strathclyde Animal Welfare and Ethical Review Body. Animal studies are reported in compliance with the ARRIVE guidelines (Kilkenny et al., 2010) and with the recommendations made by the *British Journal of Pharmacology*. The Strathclyde Biological Protection Unit is a conventional unit which undertakes FELASA quarterly health monitoring. Male Sprague–Dawley rats (10–12 week old; 250–300 g), from an in‐house colony, were used for the study. Sprague–Dawley rats are a widely used experimental model with a wealth of background information to aid interpretation of results. The animals were housed three per cage, and the cage type was North Kent Plastic model RC2F with nesting material “Sizzle Nest.” A 12:12 light dark cycle was used with a temperature range of 19–23°C (set point 21°C) and humidity levels between 45% and 65%. Animals had free access to fresh water and SDS diet RM1 (rodent maintenance). The enrichment in the cages was aspen wood chew sticks and hanging huts.

All experiments used first‐ or second‐order mesenteric arteries isolated from rats killed by either CO_2_ or by injection with pentobarbital; no differences in the results were observed with either method. Controls and experimental treatments were carried out in the same tissue, so blinding and randomization were not used.

### 2.2 High‐resolution imaging of endothelial Ca^2+^ signalling

Arteries (diameter of 200–230 μm) were isolated from the mesenteric bed, cleaned, then cut and pinned flat *en face* (endothelial side facing up) on a Sylgard block. The endothelium was then preferentially loaded with Cal‐520/AM (5 μM; with 0.02% Pluronic F‐127) in physiological saline solution (PSS) at 37°C for 30 min (Wilson, Lee, & McCarron, 2016; Wilson, Saunter, Girkin, & McCarron, 2016). Following incubation, arteries were gently washed with PSS, and the Sylgard blocks were placed face down on a custom flow chamber. Arteries were then continuously perfused with PSS at 1.5 ml·min^−1^ using a syringe pump. Endothelial Ca^2+^ activity was stimulated by swapping the PSS syringe for one containing ACh (100 nM) or GSK1016790A (GSK, 20 nM). GSK1016790A is a selective TRPV4 channel agonist and evokes Ca^2+^ influx in wild type but not TRPV4 channel knockout mice (Mannaa et al., 2018; Sonkusare et al., 2012).

In experiments examining the effects of various pharmacological agents on stimulated endothelial Ca^2+^ signalling, drugs were added to the perfusate and remained thereafter. Images were acquired at 10 Hz using an inverted fluorescence microscope (Eclipse TE300, Nikon, Tokyo, Japan) equipped with a 40× objective (S Fluor, Nikon, Tokyo, Japan, NA = 1.3) and an electron‐multiplying charge‐coupled device camera (iXon Life; Andor Technology Limited, Belfast, Northern Ireland, UK) or on an upright epi‐fluorescence microscope (FN‐1, Nikon, Tokyo, Japan) equipped with a 60× objective (CFI Fluor, Nikon, Tokyo, Japan, NA = 1.0). Cal520/AM was excited at 488 nm using a monochrometer (Polychome IV, TILL Photonics, Graefelfing, Germany) or an LED illumination system (PE‐300Ultra, CoolLED, Andover, UK).

In some experiments, the endothelium was loaded with Cal‐520 (5 μM) and a membrane permeant, caged IP_3_ (5 μM), and the Ca^2+^ response to local photolysis of caged IP_3_ examined. In these experiments, the endothelium was imaged as above, and a xenon flash lamp (Rapp Optoelecktronic, Germany) was used to uncage IP_3_ (Olson, Chalmers, & McCarron, 2012; Olson, Sandison, Chalmers, & McCarron, 2012; Wilson et al., 2019).

### 2.3 Extraction and analysis of endothelial Ca^2+^ signals

Endothelial Ca^2+^ signals were extracted automatically from fluorescence recordings using custom written Python software (RRID:SCR_001658; Lee et al., 2018; Wilson, Lee, & McCarron, 2016; Wilson, Saunter, et al., 2016). In brief, cellular regions of interest (ROIs) were first generated from average‐intensity projections of each time series recording. Intensity projections were sharpened (un‐sharp mask filter) and thresholded, generating ROIs that encompassed the majority of each cell's area. To facilitate comparisons in paired experiments, for example, where Ca^2+^ activity was recorded before and after pharmacological inhibition, ROIs were aligned and tracked across separate image acquisitions. Only cells that remained within the field of view for all recordings were included.

Cellular Ca^2+^ signals were extracted by averaging fluorescence intensity within each of the ROIs, for each frame of the image stack. Raw fluorescence signals (F) were expressed as fractional changes in fluorescence intensity (F/F_0_) by dividing each intensity value by the average intensity of a 100‐frame period exhibiting the least activity/noise (F_0_, red in Figure S1B). The F_0_ period was determined automatically by calculating, in series, the derivative of the signal, the rolling (100‐frame) SD of the derivative, and the rolling (100‐frame) summation of the rolling SD. The minimum value of the rolling summation indicates the centre of the portion of the signal with the least activity/noise.

The Ca^2+^ response to activation of TRPV4 channels contained two main components: a “slow” persistent Ca^2+^ elevation rise in the baseline Ca^2+^ levels and fast intracellular Ca^2+^ waves. To determine the underlying mechanisms, we isolated the two components from each F/F_0_ trace. The slow persistent Ca^2+^ elevation (baseline) component of each signal was extracted by applying an asymmetric least squares (ALS) smoothing function (Figure S1C–E; Eilers & Boelens, 2005). Signalling metrics describing this slow persistent Ca^2+^ elevation (amplitude; time of activation, 10–90% rise time) were then calculated from a fitted sigmoidal curve (Figure S1C–E). Cells were considered to exhibit a persistent Ca^2+^ elevation if the slow F/F_0_ component rose by more than 10‐fold the SD of the baseline noise. The propagating Ca^2+^ wave (fast) component of each signal was extracted by dividing each F/F_0_ trace by the ALS‐smoothed signal. This procedure effectively flattens the baseline and removes any slow drift from the signal (Figure S1C–E). Peaks in each Ca^2+^ signal arising from the waves were then detected automatically, using a zero‐crossing detector on derivative F/F_0_ traces (Lee et al., 2018; Wilson, Lee, & McCarron, 2016). Various signal metrics (number of peaks, peak amplitudes, peak durations, 10–90% rise time, and 90–10% fall time) were extracted from the corresponding ALS‐smoothed F/F_0_ trace. Cells were considered to exhibit Ca^2+^ wave activity if the F/F_0_ component exhibited at least one event with an amplitude more than 10‐fold the SD of the baseline noise.

In some experiments, Ca^2+^ signals were calibrated using $\left[{\mathrm{Ca}}^{2+}\right]=K_{D}\frac{F-F_{\min}}{F_{\max}-F}$, where *K*_D_ = 320 nM. *F*_max_ was determined at the end of the experiment by the addition of ionomycin (1 μM) and *F*_min_ the signal in the absence of light to the camera.

### 2.4 Measurement of vascular reactivity

To assess the endothelium dependence of contractile and dilation responses, vascular reactivity to various agonists were assessed in *en face* artery preparations. This technique permits simultaneous visualization of the endothelium and assessment of functional responses (contraction and dilation) and was used so that we could confirm the removal of the endothelium as required.

Arteries of 200–230 μm diameter were loaded with Cal‐520/AM as above, except that they were pinned to the bottom of a custom, Sylgard‐coated flow chamber. This flow chamber was designed to facilitate the perfusion of solutions (1.5 ml·min^−1^) when mounted on an upright microscope (FN‐1, Nikon, Tokyo, Japan). Handling and pinning of the arteries were restricted to the outermost corners, leaving the central portion of the vessels with intact endothelium and able to contract/dilate freely.

Following incubation, arteries were gently washed with PSS and left to equilibrate for 30 min in PSS (continuously perfused 1.5 ml·min^−1^ using a syringe pump). Contraction was stimulated by swapping the PSS for one containing phenylephrine. The concentration of phenylephrine was titrated to contract arteries to ~20% of resting diameter. Relaxation was stimulated by changing the phenylephrine‐containing syringe for one containing phenylephrine (at the same concentration) and either ACh (100 nM), GSK1016790A (GSK, 20 nM), or sodium nitroprusside (SNP, 100 μM). Contractile and dilator responses were assessed before and after (paired) various treatments in the same artery, as described in the text. After treatment, antagonists were continuously present in the PSS. The removal of the endothelium was achieved by gentle mechanical disruption with a human hair that was superglued onto a pipette tip. Endothelium removal was confirmed by the absence of endothelial cells stained with the Ca^2+^ indicator Cal‐520/AM. After the removal of the endothelium, arteries were left to equilibrate for 60 min.

Images were acquired at 10 or 5 Hz (consistent within each experimental protocol) using an upright fluorescence microscope equipped with a 16× objective lens (0.8 NA; Nikon, Tokyo, Japan) and large format (1,024 × 1,024; 13‐μm pixels) back‐illuminated electron‐multiplying charge‐coupled device camera (iXon 888; Andor, Belfast, UK) and stored for offline analysis. An edge‐detection algorithm (Lawton et al., 2019) was used to track the width of arteries in image recordings. The algorithm extracts an intensity profile along a scanline orientated perpendicular to the longitudinal axis of the vessel. Each intensity profile was smoothed using a 251‐point, fifth‐order Savitzky–Golay filter, and the first‐order derivative calculated. The edges of the artery correspond to the maxima and minima of the first‐order derivative, which were identified and tracked using a zero‐crossing detector. The width of the artery equates to the unfolded circumference of the intact vessel and was converted to the equivalent diameter. To control for variation in the resting diameters, summary contractile response data are expressed as the percentage reduction from resting diameter,while relaxation responses are expressed as the percentage increase in diameter compared to resting diameter (Figure S2).

### 2.5 Matching arterial tone across experimental conditions

Following the removal of the endothelium, arteries were significantly more sensitive to phenylephrine. The concentration of phenylephrine was titrated to achieve a level of tone that was comparable to that achieved in the presence of a functional endothelium (Figure S2A). In the presence of cyclopiazonic acid (CPA), Phenylephrine‐induced contractions were transient, and it was not possible to achieve a stable contraction. Therefore, assessing the effect of store depletion on TRPV4‐induced dilation was not feasible. Instead, vessels were pretreated with GSK and CPA, and the magnitude of the transient contraction induced by phenylephrine was assessed. Furthermore, after depletion of Ca^2+^ stores using CPA, a significantly higher concentration of phenylephrine was required to generate comparable levels of vascular tone (Figure S2B).

### 2.6 Pressure myography

Diameter measurements of pressurized arteries were performed using the VasoTracker open source pressure myograph system (system (RRID:SCR_017233; Lawton et al., 2019). Mesenteric arteries were dissected free from surrounding tissue, cleaned of connective tissue, and mounted on similar sized glass pipettes in a VasoTracker pressure myograph chamber. Arteries were pressurized to 70 mmHg, checked for leaks, and left to equilibrate for 30 min in pre‐warmed (37°C) circulating PSS, with intraluminal flow (~100 μl·min^−1^) established by a 10 cmH_2_O pressure gradient.

Artery diameter was monitored using an sCMOS camera (DCC1545M, Thorlabs, New Jersey, USA) and recorded by VasoTracker acquisition software. Arteries were contracted using phenylephrine (~500 nM) to ~80% of resting diameter, which was added to the perfusion solution. All other drugs (e.g., ACh and GSK) were applied intraluminally. Summary relaxation data are expressed as the percentage increase in diameter compared to resting diameter.

### 2.7 Materials

Cal‐520/AM was obtained from Abcam (Cambridge, MA, USA). Caged‐IP_3_ (caged‐IP_3_ 4,5‐dimethoxy‐2‐nitrobenzyl) was obtained from Sichem (Germany). Pluronic F‐127 was obtained from Invitrogen (Carlsbad, CA, USA). U73343 was obtained from Tocris (St Louis, MO, USA). All other drugs and chemicals were obtained from Sigma (St Louis, MO, USA). GSK, HC067047, CPA, U73122, U73343, 2‐aminoethoxydiphenyl borate (2‐APB), and ionomycin were dissolved in DMSO and diluted to working concentration in PSS such that the total volume of DMSO was less than or equal to 0.1%. All other drug stocks were dissolved in water. The PSS consisted of the following (mM): 145 NaCl, 4.7 KCl, 2.0 MOPS, 1.2 NaH_2_PO_4_, 5.0 glucose, 0.02 EDTA, 1.17 MgCl_2_, and 2.0 CaCl_2_ (adjusted to pH 7.4 with NaOH). In experiments using a Ca^2+^‐free PSS, Ca^2+^ was substituted with Mg^2+^ on an equimolar basis, and EGTA (1 mM) was included. All solutions were freshly prepared each day.

### 2.8 Data and statistical analysis

The data and statistical analysis in this study comply with the recommendations of the British Journal of Pharmacology on experimental design and analysis in pharmacology The *n* vales shown are the numbers of biological replicates (number of animals). For each replicate, a single field of endothelial cells (Ca^2+^ imaging experiments) or a single artery segment (vascular reactivity studies) was studied. Summary data are presented graphically as paired mean responses, or as the grand mean ± SEM of *n* biological replicates. Two‐tailed, paired t test (for paired observations), independent two‐sample *t* tests or repeated measures one‐way ANOVA with multiple comparisons were used as indicated in the text. All statistical analyses were performed using GraphPad Prism version 6.0 (RRID:SCR_002798; GraphPad Software, La Jolla, CA, USA). A *P* value of <.05 was considered statistically significant.

### 2.9 Nomenclature of targets and ligands

Key protein targets and ligands in this article are hyperlinked to corresponding entries in http://www.guidetopharmacology.org, the common portal for data from the IUPHAR/BPS Guide to PHARMACOLOGY (Harding et al., 2018), and are permanently archived in the Concise Guide to PHARMACOLOGY 2017/18 (Alexander, Christopoulos et al., 2017; Alexander, Fabbro et al., 2017; Alexander, Peters et al., 2017; Alexander, Striessnig et al., 2017).

## 3 RESULTS

### 3.1 Characteristics of ACh‐ and GSK‐evoked Ca^2+^ signals

**Figure 1:**
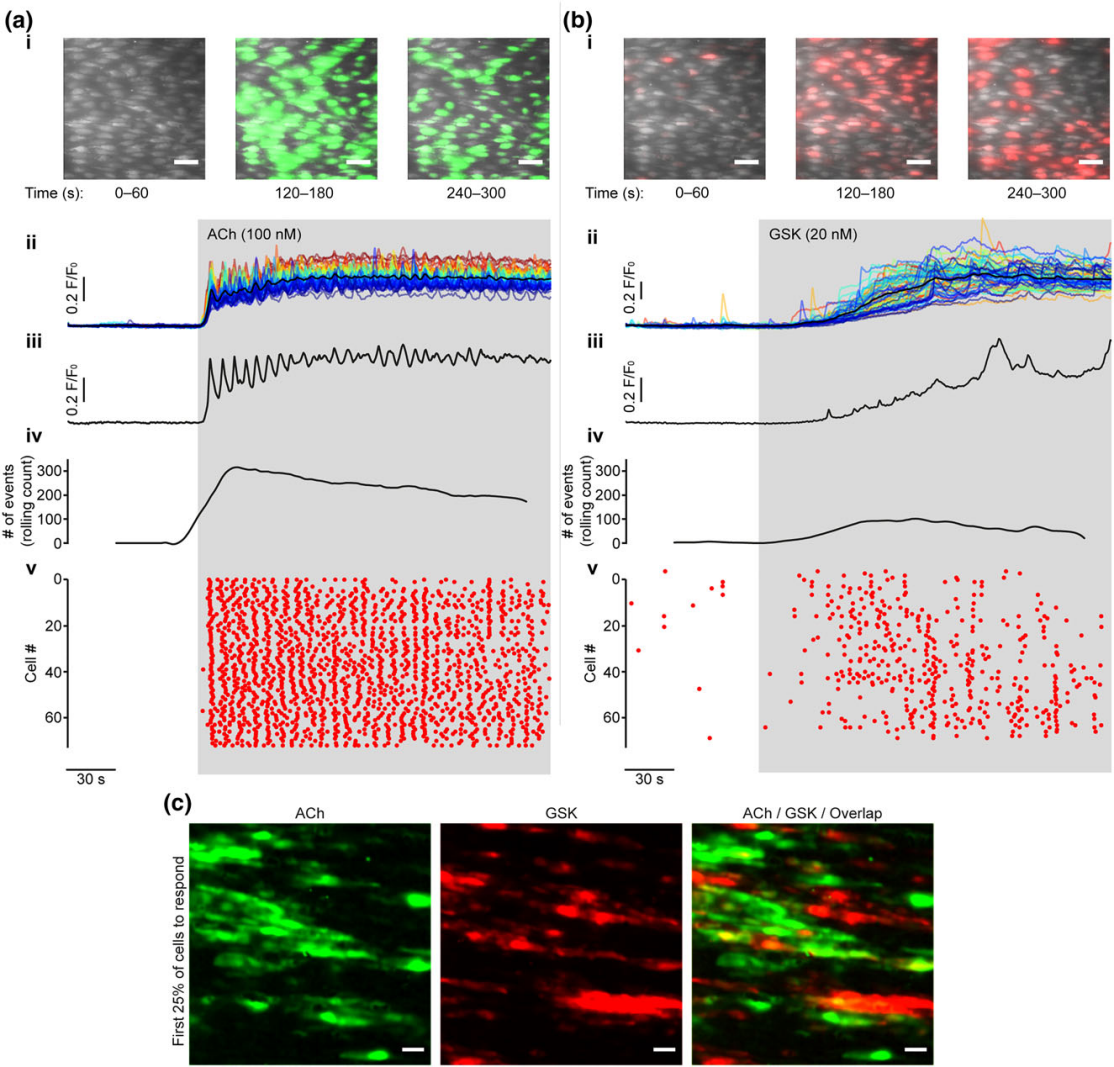
ACh and GSK1016790A stimulate different Ca^2+^ signalling patterns. (a, b) Ca^2+^ dynamics visualized in a single en face endothelial preparation stimulated with ACh (a), and GSK1016790A (GSK, b). Panels show: (i) composite images illustrating endothelial Ca^2+^ activity over the time course of the experiment, scale bars = 50 μm; (ii) whole‐cell Ca^2+^ traces (F/F_0_) for all cells shown in the corresponding panel in (i) ranked and coloured according to the magnitude of the signal (dark blue, low; dark red, high); (iii) a representative Ca^2+^ trace from a single cell; (iv) a rolling summation (30 s) of the number of Ca^2+^ events (peaks) across the‐field of‐view; and (v) a rastergram display of Ca^2+^ activity, where each dot represents the occurrence of a peak in the Ca^2+^ response from each cell. (c) Representative images showing endothelial cells that were most sensitive (first 25% of endothelial cells to respond) to application of either ACh (green) or GSK (red). On average, 32.6% of cells that responded to one agonist also responded to the other (indicated by yellow in right most panel; n = 3). Scale bars = 20 μm

To determine if activation of TRPV4 channels in endothelial cells evoked Ca^2+^ release from the internal store, endothelial cells were loaded with the fluorescent Ca^2+^ indicator Cal520/AM and activated with ACh (100 nM) or the TRPV4 channel agonist GSK (20 nM). Ca^2+^ activity was then visualized in the fields of ~100 endothelial cells. Cellular responses were analysed individually (Figures 1 and S1).

**Figure 2:**
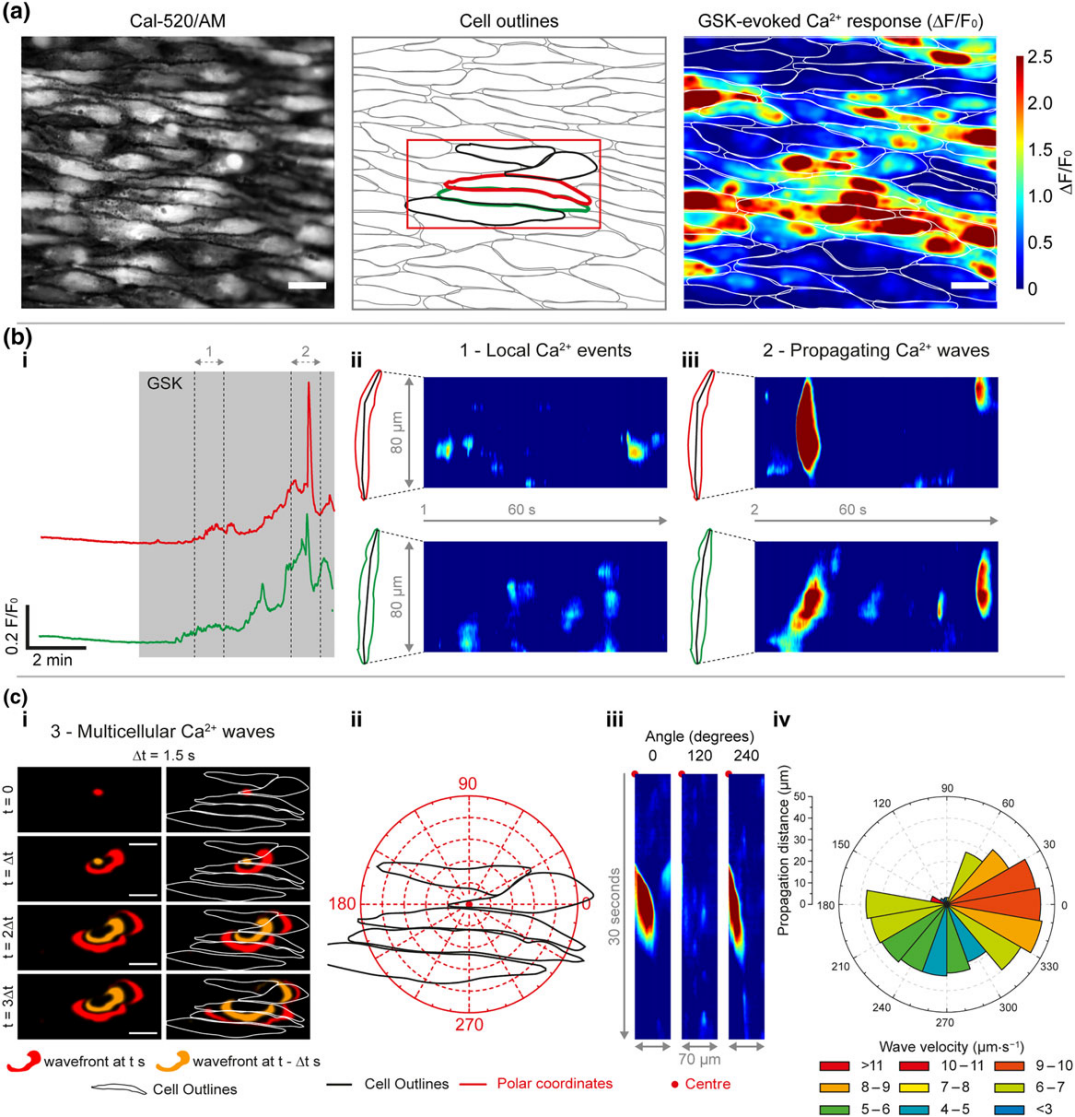
Activation of TRPV4 channels stimulates local and propagating Ca^2+^ events. (a) Representative Ca^2+^ image (left), cell outlines (middle), and ΔF/F_0_ maximum intensity projection (right) illustrating the endothelial Ca^2+^ response to activation of TRPV4 channels with GSK1016790A (GSK; 20 nM). The ΔF/F_0_ image shows an increase in Ca^2+^ across the field of endothelial cells. (b) GSK stimulates local Ca^2+^ events and propagating Ca^2+^ waves. Panels show: (i) Ca^2+^ traces illustrating the effect of GSK on whole‐cell Ca^2+^ levels from cells indicated by the coloured outlines in (a); (ii) line scans (kymographs) showing GSK‐evoked local Ca^2+^ events (1) and propagating Ca^2+^ waves (2). Kymographs in (ii) correspond to the times indicated by dashed lines in (i) and were generated from F/F_Δt_ movies, where F_Δt_ is the running (20 s) baseline. (c) Ca^2+^ waves propagate across multiple cells. Panels show: (i) time series of the active front (determined by sequential subtraction) of a multicellular Ca^2+^ wave that spreads across four endothelial cells; (ii, iii) polar coordinate system (ii), example F/F_Δt_ radial line scans (iii, 20° integration angle), and polar plot (iv) showing the angular dependence of propagation distance/velocity for the depicted Ca^2+^ event. Data in (c) are shown in Movie S2. All scale bars = 20 μm

Ca^2+^ responses evoked by ACh and GSK were clearly different (Figure 1). ACh evoked a rapid elevation in intracellular Ca^2+^ which was followed by asynchronous oscillations across the field of view (Figure 1; Mumtaz et al., 2011). Activation of TRPV4 channels resulted in heterogeneous Ca^2+^ responses (Figure 1c, Movie S1, and Figure S3) that consisted of several distinct phases (Figures 1 and 2). The response to GSK was, initially, small localized Ca^2+^ spikes which led to a slowly increasing global cytoplasmic Ca^2+^ concentration (Figures 2b and S4). The GSK‐evoked, global Ca^2+^ elevation eventually plateaued, but the time taken to plateau was significantly longer when compared to ACh (Figure 1; 55.9 ± 6.4 s for GSK; 16.7 ± 4.7 s for ACh; *n* = 6). Finally, large propagating Ca^2+^ waves developed (Figure 2b,c and Movies S1 and S2). These waves propagated within and between cells at a constant velocity of ~5 to 15 μm·s^−1^ (Figure 2b,c, Movie S2, and Figure S5). GSK‐evoked intracellular Ca^2+^ waves were significantly lower in frequency than those for ACh (0.05 ± 0.01 Hz for GSK; 0.22 ± 0.03 Hz for ACh; *n* = 6).

These results suggest there are at least major two components of the Ca^2+^ signal arising from activation of TRPV4 channels: (a) initial local signals which lead to a slow global rise in Ca^2+^ and (b) large fast propagating Ca^2+^ waves.

We next sought to examine the mechanisms that give rise to the GSK‐evoked Ca^2+^ signals. We first confirmed the reproducibility of GSK‐evoked responses (20 nM) in the same preparation so that a paired experimental design could be used to examine the effects of blockers. There was no significant difference in any of the Ca^2+^ signal parameters measured (number of peaks, peak amplitudes, peak durations, 10–90% rise time, and 90–10% fall time) on repeated applications of GSK (Figure S3).

### 3.2 Ca^2+^ influx via TRPV4 channels is required to initiate endothelial Ca^2+^signals

To examine the mechanisms underlying each component of the GSK‐evoked Ca^2+^ signal, we developed methods to extract the slow global rise in Ca^2+^ and the propagating Ca^2+^ waves (fast) component from raw Ca^2+^ signals in each cell (Figure S1).

**Figure 3:**
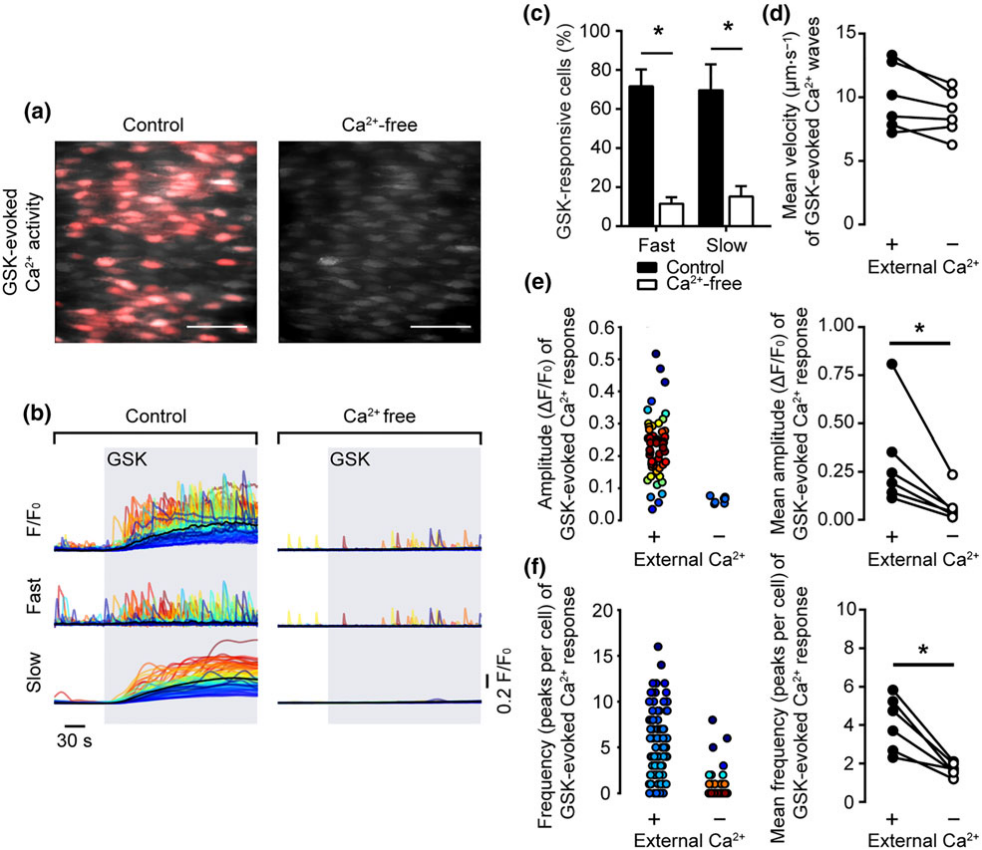
The removal of extracellular Ca^2+^ abolishes GSK‐evoked Ca^2+^ signalling. (a) Composite images showing GSK1016790A (GSK)‐evoked Ca^2+^ activity in a single field of native mesenteric artery endothelial cells before (left, control) and after (right, Ca^2+^‐free) the removal of external Ca^2+^. Images show basal Cal‐520/AM fluorescence (grey) with Ca^2+^ activity overlaid (red). Scale bars = 50 μm. (b) GSK‐evoked (20 nM; grey box) cellular Ca^2+^ signals (F/F_0_) in the presence (left) and absence (right) of extracellular Ca^2+^. F/F_0_ signals (top) were decomposed into fast (middle) and slow (bottom) components. (c) Summary bar chart showing the percentage of cells that exhibited propagating Ca^2+^ waves (fast) and slow global Ca^2+^ elevations (slow). (d–f) Paired summary data showing the effect of external Ca^2+^ inhibition on Ca^2+^ wave propagation velocity (d), peak amplitude (ΔF/F_0_, e), and the number of peaks per cell (f). The left plots in (e and f) are scatter plots showing the mean Ca^2+^ event amplitude, or oscillation frequency, within each cell visualized in the experiment shown in panels (a and b). Individual data points are coloured (from blue, low to red, high) according to the density (i.e., occurrence) of particular values. *P < .05, significantly different as indicated; paired Student's t test (n = 6)

To determine if each component is dependent on Ca^2+^ entry, we first performed experiments using a Ca^2+^‐free PSS (Figure 3a,b). The removal of extracellular Ca^2+^ significantly decreased the percentage of cells exhibiting “slow” global Ca^2+^ elevations and “fast” propagating waves in response to GSK (Figure 3c). The velocity of residual Ca^2+^ waves was similar to those occurring in the presence of external Ca^2+^ (Figure 3d; 10.0 ± 1.1 μm·s^−1^ for control; 8.8 ± 0.7 μm·s^−1^ for Ca^2+^‐free; *n* = 5). However, removal of external Ca^2+^ significantly reduced the amplitude (Figure 3e; 0.28 ± 0.11 ΔF/F_0_ for control; 0.02 ± 0.10 ΔF/F_0_ for Ca^2+^‐free; *n* = 6) and the frequency of occurrence (Figure 3f; 4.1 ± 0.6 peaks per cell for control; 1.7 ± 0.1 peaks per cell for Ca^2+^‐free; *n* = 6) of these residual propagating Ca^2+^ waves.

These results suggest that Ca^2+^ influx across the plasma membrane is essential for the slow global increase in Ca^2+^ and for the propagating Ca^2+^ waves evoked by GSK.

**Figure 4:**
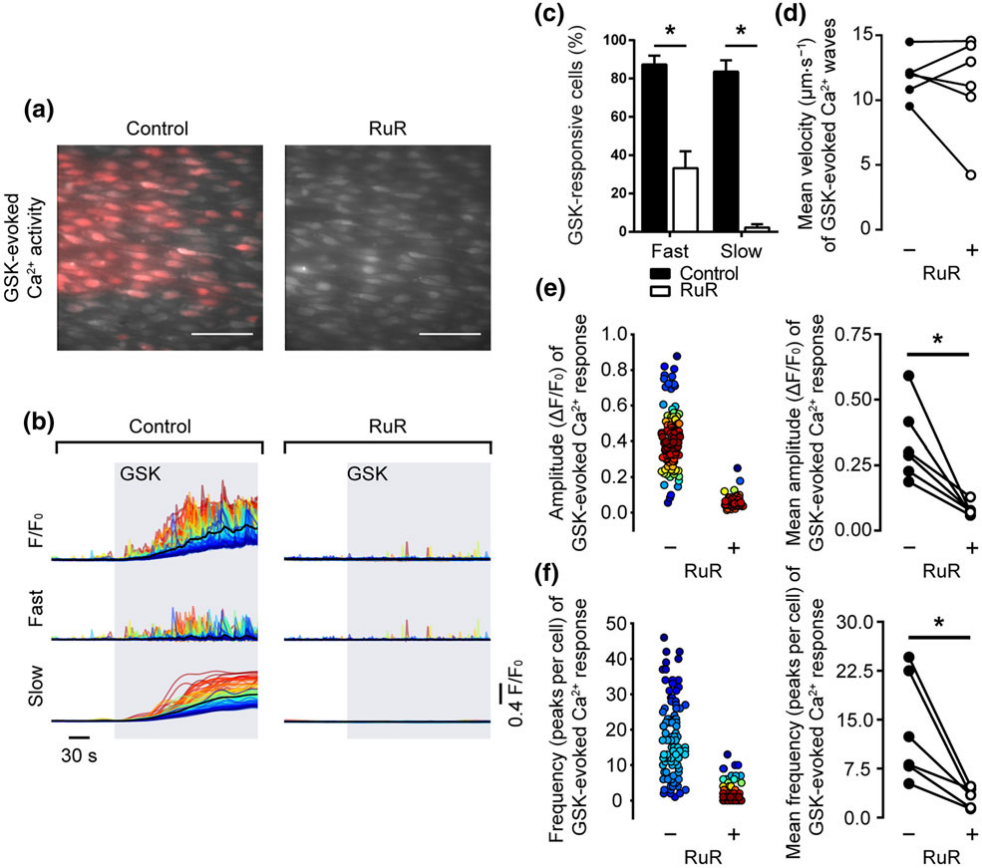
Inhibition of TRPV channels with ruthenium red inhibits propagating Ca^2+^ waves and slow global Ca^2+^ elevations evoked by GSK1016790A. (a) Composite images showing GSK1016790A (GSK)‐evoked (20 nM) Ca^2+^ activity in the absence (left) and presence (right) of the broad‐spectrum TRPV channel antagonist ruthenium red (RuR; 5 μM, 20 min) in the same native mesenteric artery endothelial cells. Images show basal Cal‐520/AM fluorescence (grey) with Ca^2+^ activity overlaid (red). Scale bars = 50 μm. (b) Raw F/F_0_ (top), fast (middle), and slow (bottom) components of the GSK‐evoked (20 nM; grey box) Ca^2+^ signal in the absence (left) and presence (right) of RuR. (c) Summary bar chart illustrating the effect of RuR on the percentage of cells exhibiting propagating Ca^2+^ waves and slow global Ca^2+^ signal components. (d–f) Paired summary data showing the effect of TRPV channel inhibition on Ca^2+^ wave propagation velocity (d), peak amplitude (ΔF/F_0_, e), and the number of peaks per cell (f). The left plots in (e–f) are scatter plots showing the mean Ca^2+^ event amplitude, or oscillation frequency, within each cell visualized in the experiment shown in panels (a and b). Individual data points are coloured (from blue, low to red, high) according to the density (i.e., occurrence) of particular values. *P < .05, significantly different as indicated; paired Student's t test (n = 6)

**Figure 5:**
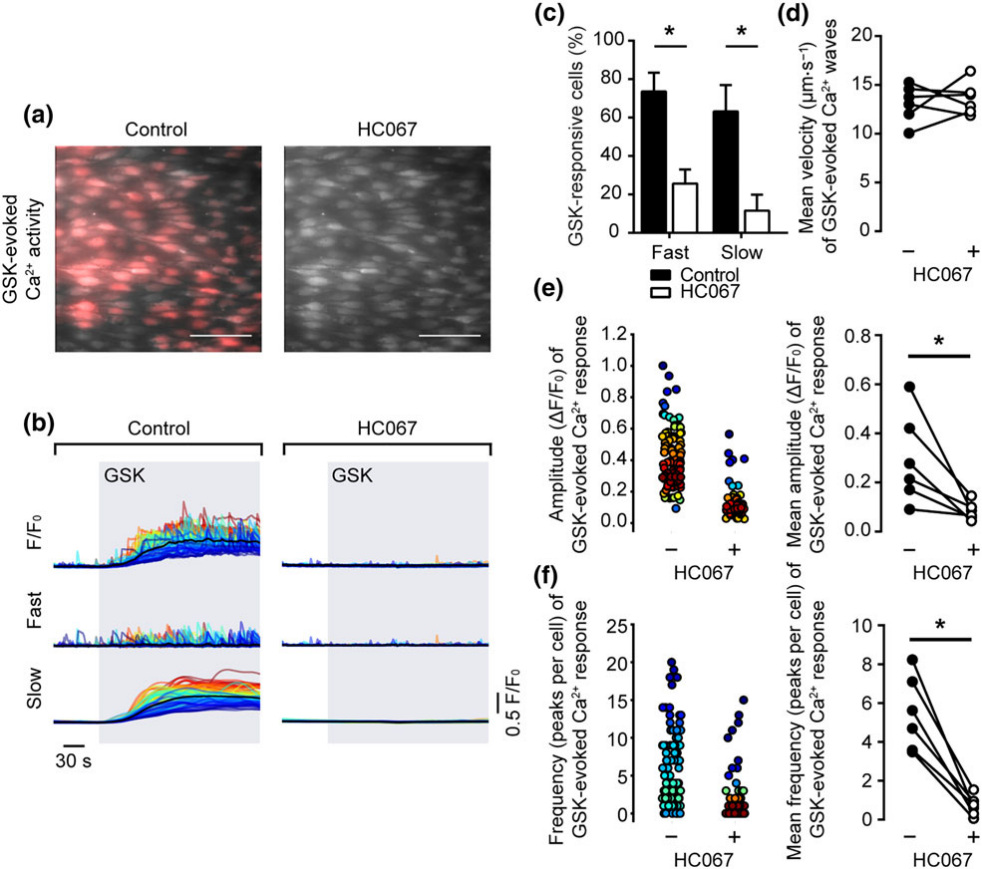
Inhibition of TRPV4 channels with HC067047 inhibits both propagating Ca^2+^ waves and slow global Ca^2+^ elevations evoked by GSK1016790A. (a) Composite images showing GSK1016790A (GSK)‐evoked (20 nM) Ca^2+^ activity in the absence (left) and presence (right) of the broad‐spectrum TRPV channel antagonist HC067047 (HC067; 10 μM, 20 min) in the same native mesenteric artery endothelial cells. Images show basal Cal‐520/AM fluorescence (grey) with Ca^2+^ activity overlaid (red). Scale bars = 50 μm. (b) Raw F/F_0_ (top), fast (middle), and slow (bottom) components of the GSK‐evoked (grey box) Ca^2+^ signal in the absence (left) and presence (right) of HC067. (c) Summary bar chart illustrating the effect of HC067 on the percentage of cells exhibiting propagating Ca^2+^ waves and slow global Ca^2+^ signal components. (d–f) Paired summary data showing the effect of TRPV4 channel inhibition on Ca^2+^ wave propagation velocity (d), peak amplitude (ΔF/F_0_, e), and the number of peaks per cell (f). The left plots in (e–f) are scatter plots showing the mean Ca^2+^ event amplitude, or oscillation frequency, within each cell visualized in the experiment shown in panels (a and b). Individual data points are coloured (from blue, low to red, high) according to the density (i.e., occurrence) of particular values. *P < .05, significantly different as indicated; paired Student's t test (n = 6)

To test whether or not the slow global Ca^2+^ rise and propagating Ca^2+^ waves evoked by GSK arose from the activation of TRPV4 channels, we pre‐incubated endothelial cells with the non‐selective TRPV channel antagonist, ruthenium red (RuR; 5 μm, 20 min; Figure 4a,b). RuR significantly reduced the percentage of cells responding to GSK with slow global Ca^2+^ elevations and propagating Ca^2+^ waves (Figure 4c). Pretreatment with RuR reduced the amplitude (Figure 4e; 0.33 ± 0.06 ΔF/F_0_ for control; 0.07 ± 0.01 ΔF/F_0_ for RuR; *n* = 6) and the frequency of occurrence (Figure 4f; 13.4 ± 3.3 peaks per cell for control; 3.2 ± 0.1 peaks per cell for RuR; *n* = 6) of propagating Ca^2+^ waves but had no effect on the velocity of propagating waves (Figure 4d; 11.8 ± 0.7 μm·s^−1^ for control; 11.6 ± 1.7 μm·s^−1^ for RuR; *n* = 5). A similar reduction in GSK‐evoked Ca^2+^ signalling was observed using the selective TRPV4 channel antagonist, HC067047 (HC067; 10 μM, 20 min; Figure 5a,b). HC067 reduced the percentage of cells exhibiting slow, global elevations and the percentage exhibiting fast, propagating Ca^2+^ waves (Figure 5c). HC067 reduced the amplitude (Figure 5e; 0.29 ± 0.08 ΔF/F_0_ for control; 0.08 ± 0.02 ΔF/F_0_ for HC067; *n* = 6) and the frequency (Figure 5f; 5.5 ± 0.8 peaks per cell for control; 0.7 ± 0.2 peaks per cell for HC067; *n* = 6) of propagating Ca^2+^ waves. The velocity of residual Ca^2+^ waves was unaffected by HC067 (Figure 5d; 13.1 ± 0.8 μm·s^−1^ for control; 13.6 ± 0.7 μm·s^−1^ for HC067; *n* = 5).

Together, these results suggest that Ca^2+^ influx via TRPV4 channels is required to induce both the slow global rise component and the propagating Ca^2+^ waves induced by GSK.

### 3.3 GSK‐induced Ca^2+^waves require a replete internal Ca^2+^ store, IP_3_ synthesis, and IP_3_ receptor activation

**Figure 6:**
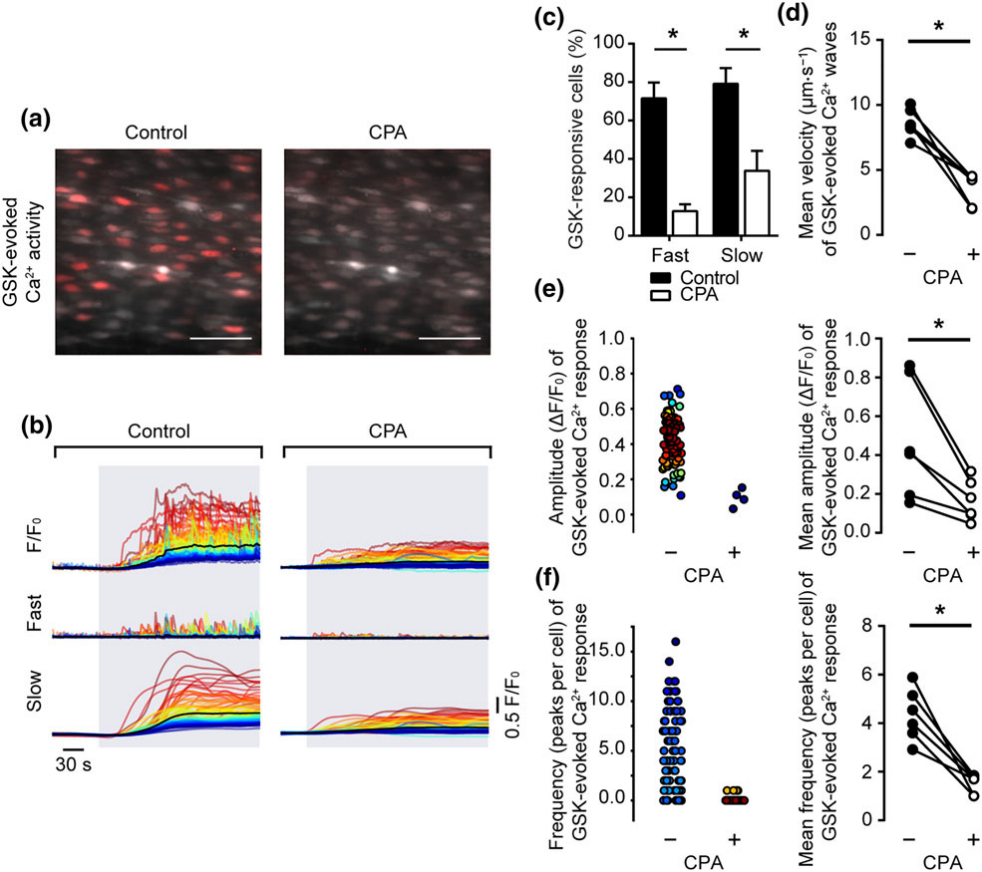
Depletion of the internal Ca^2+^ store modifies the Ca^2+^ signal induced by activation of TRPV4 channels. (a) Composite images showing GSK1016709A (GSK)‐evoked Ca^2+^ activity in the absence (left) and presence (right) of cyclopiazonic acid (CPA; 10 μM, 5 min) in the same native mesenteric artery endothelial cells. Images show basal Cal‐520/AM fluorescence (grey) with Ca^2+^ activity overlaid (red). Scale bars = 50 μm. (b) GSK‐evoked (20 nM; grey box) cellular Ca^2+^ signals in the absence and presence of CPA (left and right respectively). Original F/F_0_ traces (top) were decomposed into fast (middle) and slow (bottom) components. CPA evoked a substantial increase in basal Ca^2+^ (see Section 3). In control and in the presence of CPA, F/F_0_ was normalized to the Ca^2+^ change before GSK so that the resting values in each case were 1. CPA caused a substantial increase in resting Ca^2+^ (see text). (c) Summary bar chart illustrating the effect of CPA on the percentage of cells exhibiting propagating Ca^2+^ waves and slow global Ca^2+^ elevations to GSK. (d–f) Paired summary data showing the effect of SERCA inhibition on Ca^2+^ wave propagation velocity (d), peak amplitude (ΔF/F_0_, e), and the number of peaks per cell (f). The left plots in (e–f) are scatter plots showing the mean Ca^2+^ event amplitude, or oscillation frequency, within each cell visualized in the experiment shown in panels (a and b). Individual data points are coloured (from blue, low to red, high) according to the density (i.e., occurrence) of particular values. *P < .05, significantly different as indicated; paired Student's t test (n = 6)

Having confirmed a role for TRPV4‐mediated Ca^2+^ influx in GSK‐stimulated Ca^2+^ signals, we next investigated the contribution of the internal Ca^2+^ store using the sarcoplasmic/endoplasmic reticulum Ca^2+^ ‐ATPase (SERCA) inhibitor, CPA (Figure 6a,b). Depletion of the internal store by CPA decreased the percentage of cells exhibiting the slow global rise component of the GSK‐evoked Ca^2+^ signal and the percentage of cells in which propagating Ca^2+^ waves occurred (Figure 6c). CPA significantly inhibited the wave propagation speed (Figure 6d; 8.6 ± 0.4 μm·s^−1^ for control; 3.6 ± 0.5 μm·s^−1^ for CPA; *n* = 6), amplitude (Figure 6e; 0.48 ± 0.12 ΔF/F_0_ for control; 0.17 ± 0.04 ΔF/F_0_ for CPA; *n* = 6), and frequency (Figure 6f; 4.3 ± 0.4 peaks per cell for control; 1.5 ± 0.2 peaks per cell for CPA; *n* = 6) of Ca^2+^ waves. The amplitude of the slow global Ca^2+^ rise initially appeared to be reduced by CPA (Figure 6b). However, this apparent decrease appears to occur in large part because of the increase in baseline Ca^2+^ evoked by CPA and the non‐linear relationship between Ca^2+^ concentration and F/F_0_ value. When the signals were calibrated (see Section 2), the mean Ca^2+^ concentration increase evoked by GSK was 123 ± 75 nM, but after CPA, GSK evoked a Ca^2+^ rise of 93 ± 44 nM (*n* = 3), showing no significant effect of CPA. CPA itself evoked an increase in Ca^2+^ concentration from a resting value of 176 ± 52 to 335 ± 58 nM (*n* = 3). The elevated Ca^2+^ may account for the reduced number of cells activated by GSK (Watanabe et al., 2003). These results suggest that the internal store is required for the propagating Ca^2+^ waves.

**Figure 7:**
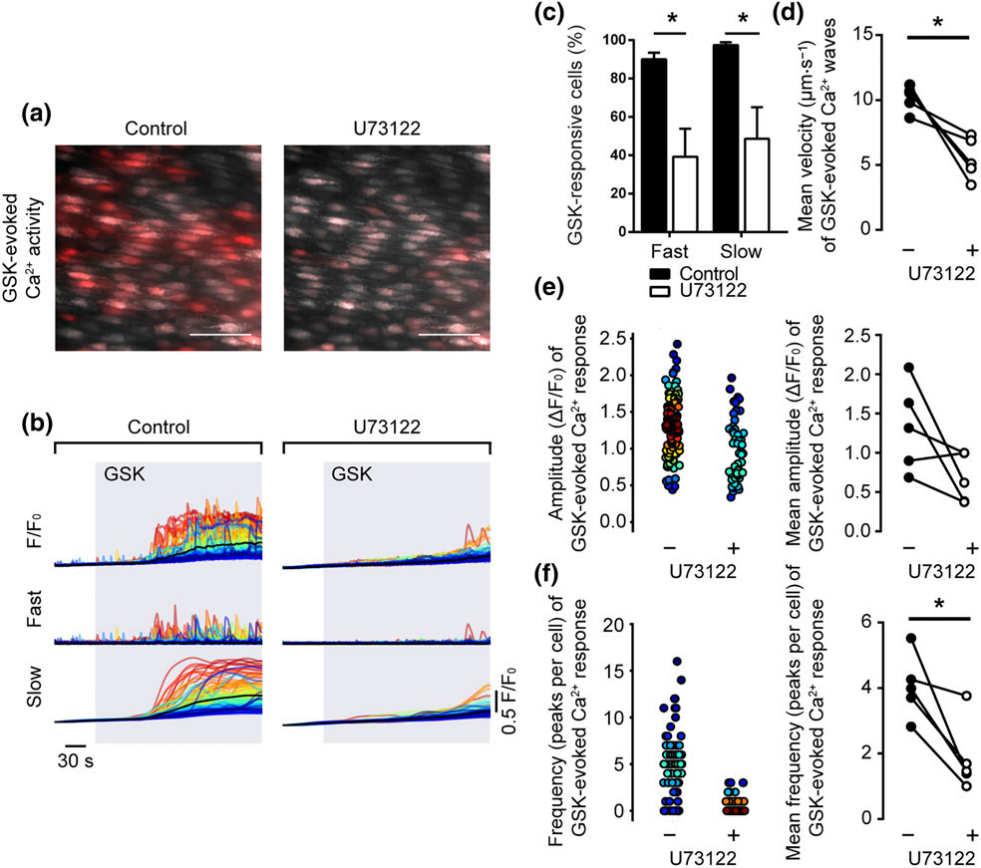
PLC inhibition reduces the slow global Ca^2+^ rises and propagating Ca^2+^ waves of the GSK‐evoked Ca^2+^ response. (a) Composite images illustrating GSK1016790A (GSK)‐evoked (20 nM) Ca^2+^ activity in the absence (left) and presence (right) of the PLC antagonist U73122 (2 μM, 10 min) in a single field of native mesenteric artery endothelial cells. Images show basal Cal‐520/AM fluorescence (grey) with Ca^2+^ activity overlaid (red). Scale bars = 50 μm. (b) GSK‐evoked F/F_0_ (top) Ca^2+^ signals, and corresponding fast (middle) and slow (bottom) signal components in the absence (left) and presence (right) of U73122. (c) Summary data showing the effect of U73122 on the percentage of endothelial cells exhibiting propagating Ca^2+^ waves (fast) and global Ca^2+^ elevations (slow). (d–f) Paired summary data showing the effect of PLC inhibition on Ca^2+^ wave propagation velocity (d), peak amplitude (ΔF/F_0_, e), and the number of peaks per cell (f). The left plots in (e–f) are scatter plots showing the mean Ca^2+^ event amplitude, or oscillation frequency, within each cell visualized in the experiment shown in panels (a and b). Individual data points are coloured (from blue, low to red, high) according to the density (i.e., occurrence) of particular values. *P < .05, significantly different as indicated; paired Student's t test (n = 5)

To explore the role of Ca^2+^ release from the internal store, we examined the contribution of IP_3_ using pharmacological inhibition of phospholipase C (PLC; Figure 7a,b). The PLC inhibitor, U73122, significantly reduced the percentage of cells activated by GSK, both for the fast propagating Ca^2+^ waves and for the slow persistent Ca^2+^ elevation (Figure 7c). Although the mean amplitude of propagating Ca^2+^ waves was not significantly different in the presence of U73122 (1.32 ± 0.25 ΔF/F_0_ for control; 0.67 ± 0.14 for U73122; *n* = 5), there was a significant reduction in the propagation velocity (10.2 ± 0.4 μm·s^−1^ for control; 5.5 ± 0.7 μm·s^−1^ for U73122; *n* = 5) and frequency of those events (Figure 7d‐f).

U73343, the inactive analogue of the PLC inhibitor U73122, had no effect on GSK‐induced Ca^2+^ signals (Figure S6). The percentage of cells exhibiting slow and propagating Ca^2+^ wave components of the GSK‐evoked signal were unaltered. Pretreatment with U73343 did not significantly alter the propagation velocity (9.4 ± 0.4 μm·s^−1^ for control; 7.7 ± 0.3 μm·s^−1^ for U73343; *n* = 5), amplitude (1.34 ± 0.26 ΔF/F_0_ for control; 1.34 ± 0.14 for U73343; *n* = 5), nor the frequency (3.5 ± 0.3 peaks/cell for control; 4.3 ± 0.6 peaks/cell for U73343; *n* = 5) of propagating Ca^2+^ waves. Moreover, although U73122 inhibits the internal store Ca^2+^ pump in colonic smooth muscle cells (Macmillan & McCarron, 2010), the blocker did not reduce IP_3_‐evoked Ca^2+^ release in endothelial cells (Figure S7).

These results suggest that the GSK‐evoked slow global rise in Ca^2+^ and propagating Ca^2+^ waves each have contributions from PLC activation.

**Figure 8:**
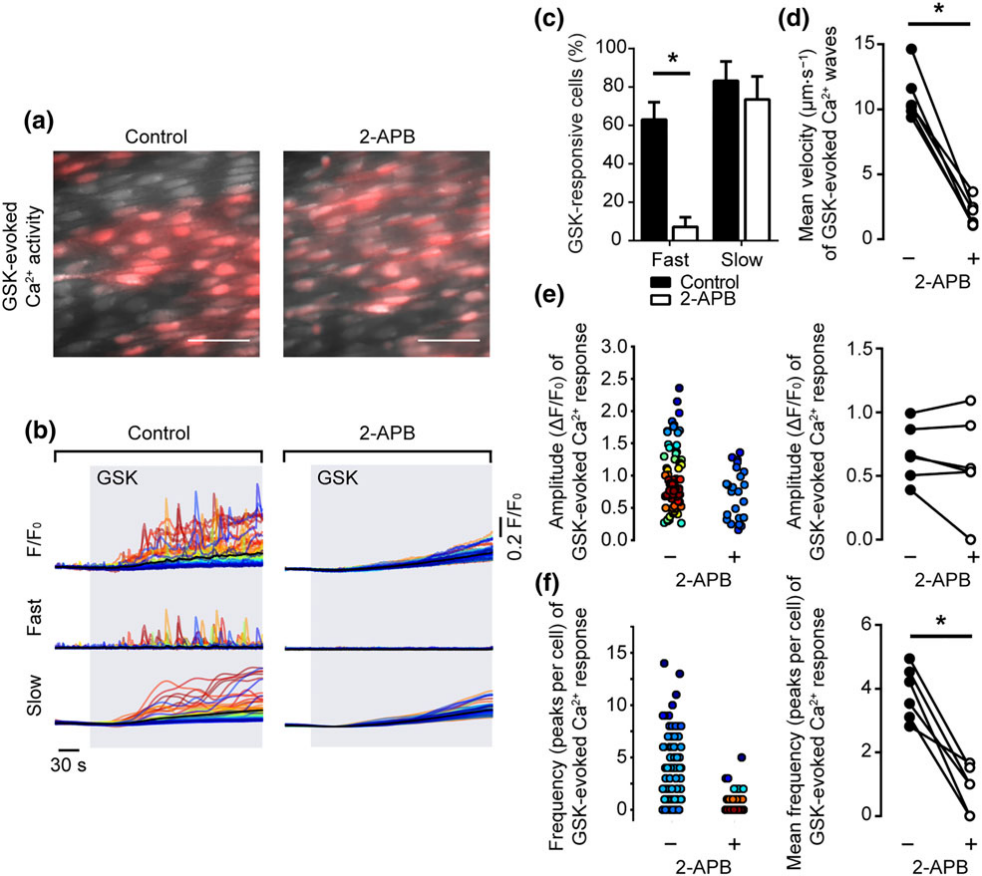
Inhibiting IP_3_ receptors prevents propagating Ca^2+^ waves but not slow global Ca^2+^ elevations induced by activation of TRPV4 channels. (a) Composite images showing Ca^2+^ activity evoked by GSK1016790A (GSK, 20 nM) in the absence (left) and presence (right) of the IP_3_R antagonist, 2‐APB (500 μM, 10 min) in a single field of native mesenteric artery endothelial cells. Images show basal Cal‐520/AM fluorescence (grey) with Ca^2+^ activity overlaid (red). Scale bars = 50 μm. (b) GSK‐evoked (grey box) cellular Ca^2+^ signals (F/F_0_; top) were decomposed into fast (middle) and slow (bottom) components. (c) Summary data illustrating the percentage of cells exhibiting propagating Ca^2+^ waves and slow global Ca^2+^ increases. (d–f) Paired summary data showing the effect of IP_3_R inhibition on Ca^2+^ wave propagation velocity (d), peak amplitude (ΔF/F_0_, e), and the number of peaks per cell (f). The left plots in (e–f) are scatter plots showing the mean Ca^2+^ event amplitude, or oscillation frequency, within each cell visualized in the experiment shown in panels (a and b). Individual data points are coloured (from blue, low to red, high) according to the density (i.e., occurrence) of particular values. *P < .05, significantly different as indicated; paired Student's t test (n = 6)

To investigate any role of IP_3_‐sensitive Ca^2+^ stores in propagating Ca^2+^ waves induced by activation of TRPV4 channels, we examined the effects of the IP_3_R antagonist, 2‐APB, on GSK‐evoked endothelial Ca^2+^ signalling (Figure 8). 2‐APB significantly reduced the percentage of cells exhibiting propagating Ca^2+^ waves but had no effect on the percentage of cells exhibiting slow global elevations in Ca^2+^ in response to GSK (Figure 8c). 2‐APB had no effect on the amplitude (0.67 ± 0.09 ΔF/F_0_ for control; 0.60 ± 0.15 ΔF/F_0_ for 2‐APB; *n* = 6) but significantly reduced propagation velocity (11.0 ± 0.8 μm·s^−1^ for control; 2.0 ± 0.4 μm·s^−1^ for 2‐APB; *n* = 5) and frequency (3.1 ± 0.3 peaks per cell for control; 0.9 ± 0.3 peaks per cell for 2‐APB; *n* = 6) of fast Ca^2+^ waves (Figure 8d–f).

To crosscheck the contribution of IP_3_Rs in the propagating Ca^2+^ waves, we used caffeine—a potent inhibitor of IP_3_Rs (Ehrlich, Kaftan, Bezprozvannaya, & Bezprozvanny, 1994; Parker & Ivorra, 1991; Saleem, Tovey, Molinski, & Taylor, 2014). Caffeine, which does not evoke Ca^2+^ release in the endothelial cells under study and inhibits Ca^2+^ release evoked by IP_3_ (Wilson et al., 2019), also blocked GSK‐evoked propagating Ca^2+^ waves (Figure S8). Interestingly, caffeine also reduced the slow global Ca^2+^ rise suggesting an effect of caffeine also on TRPV4 channels.

While Ca^2+^ influx via TRPV4 channels triggered large propagating Ca^2+^ waves from the internal Ca^2+^ store, the release and influx did not become an uncontrolled self‐regenerative process but remained under the control of Ca^2+^ influx. When activation of TRPV4 channels ceases, by washout of GSK, the propagating Ca^2+^ waves stop (Figure S9 and Movie S3), and Ca^2+^ returned towards resting values. This result demonstrates that TRPV4 channels maintain control of Ca^2+^ release from the internal Ca^2+^ store and the propagation of Ca^2+^ waves both within and between cells.

### 3.4 The functional effects of TRPV4 channels in controlling vascular tone

Collectively, our results suggest that endothelial TRPV4‐mediated Ca^2+^ influx activates Ca^2+^‐induced Ca^2+^ release at IP_3_Rs. To investigate any physiological contribution of this process, we examined vascular tone in mesenteric arteries (Figure 8) with and without a functionally intact endothelial layer.

In intact, pressurized arteries, ACh and GSK each relaxed arteries and reversed phenylephrine‐mediated contractions (Figure S10; *n* = 5). Relaxations to GSK were reversed by the selective TRPV4 channel antagonist, HC067. However, the dilations to ACh were preserved after blockade of TRPV4 channels with HC067 (Figure S10; *n* = 5). These results suggest that these channels do not contribute to ACh‐evoked relaxations in rat mesenteric arteries (see also Hartmannsgruber et al., 2007; Kohler et al., 2006; Wilson, Lee, & McCarron, 2016).

**Figure 9:**
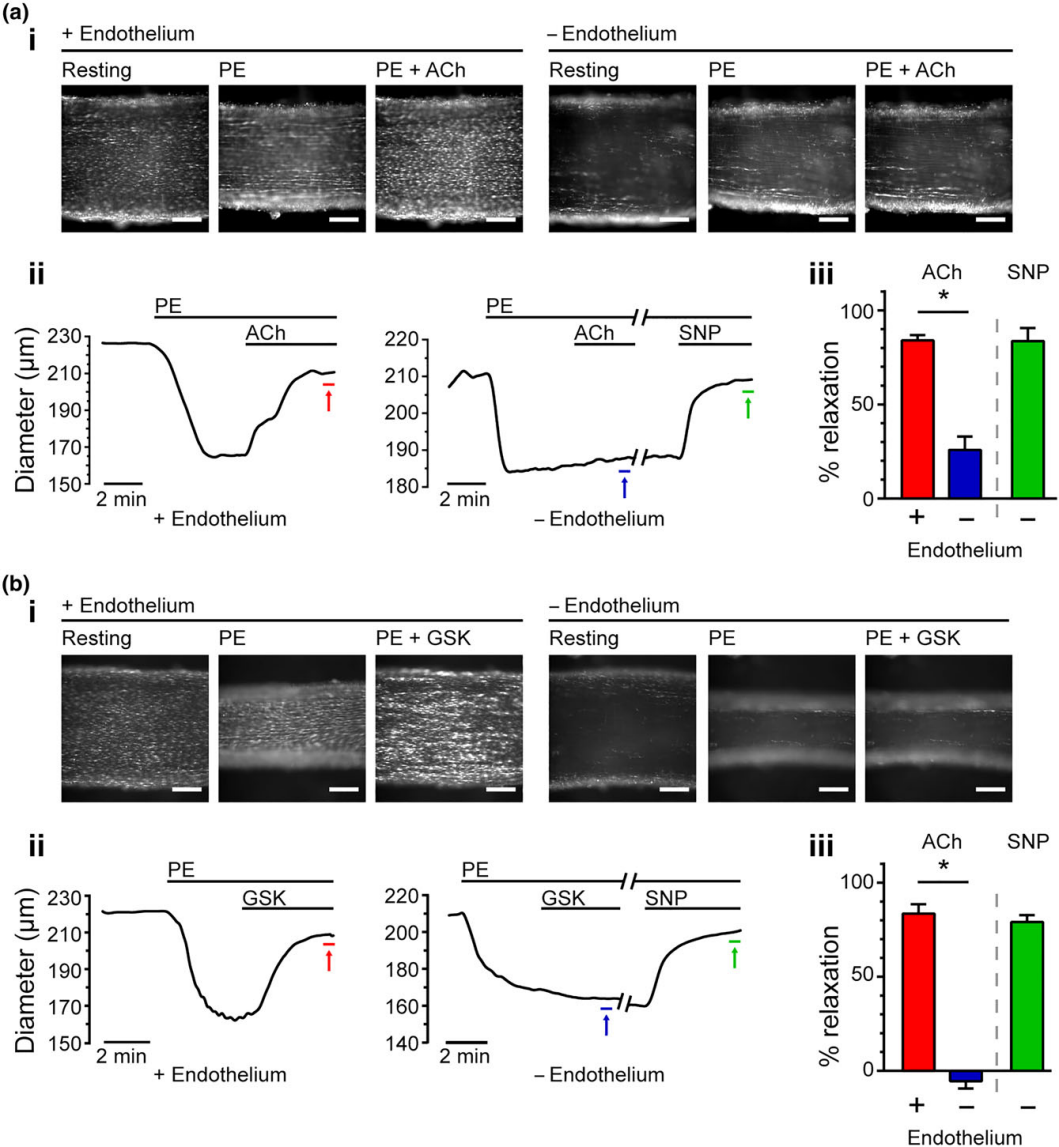
ACh and GSK1016790A both cause endothelium‐dependent vasodilation. (a and b) The vasodilator effect of ACh (a; 100 nM) and GSK1016790A (b; GSK; 20 nM) on phenylephrine (PE)‐contracted arteries before (+ endothelium) and after the mechanical removal of the endothelium (− endothelium). After the removal of the endothelium, sodium nitroprusside (SNP) was used to test endothelium‐independent vasodilation. Panels show: (i) images of an en face artery, illustrating the vessel's responses to phenylephrine and either ACh or GSK (in the continued presence of phenylephrine ); (ii) full time course of the experiments shown in the corresponding panel (i); (iii) Summary data (mean ± SEM, n = 5) illustrating the effect of the endothelium removal on vasodilator responses to the indicated agonist. Relaxations are expressed as % change from PE‐induced tone. A negative value indicates an enhancement of phenylephrine‐induced contraction. Coloured arrows in (ii) indicate the time at which relaxation to each agonist was assessed. All scale bars = 100 μm. *P < .05, significantly different as indicated; repeated measures one‐way ANOVA with multiple comparison

We next investigated the contribution of the internal Ca^2+^ store to TRPV4‐mediated relaxation evoked by GSK. In *en face* preparations (225 ± 4 μm resting diameter; *n* = 10), ACh and GSK each relaxed phenylephrine‐constricted arteries back to pre‐constricted levels (Figure 9 and Figure S2). In these same arteries, the removal of the endothelium increased the sensitivity to phenylephrine and significantly reduced relaxation to ACh (to 26 ± 7%; *n* = 5 vs. control with endothelium). The residual ACh‐evoked response may have arisen from the incomplete removal of the endothelium. The functional removal of the endothelium also prevented GSK‐evoked relaxations. Indeed, after the removal of the endothelium, GSK caused an increase in tone (Figure 9e; *n* = 5). Endothelium removal did not prevent relaxation to the endothelium‐independent vasodilator SNP (Figure 9).

These results demonstrate that vascular relaxation to GSK occurs via an endothelium‐dependent mechanism and not by acting directly on the underlying smooth muscle.

### 3.5 Store dependence of the TRPV4 channel response

We next examined the contribution of Ca^2+^‐induced Ca^2+^ release at IP_3_Rs in TRVP4 mediated relaxation. To do this, we assessed the effect of GSK on vascular reactivity before and after depletion of internal Ca^2+^ stores.

**Figure 10:**
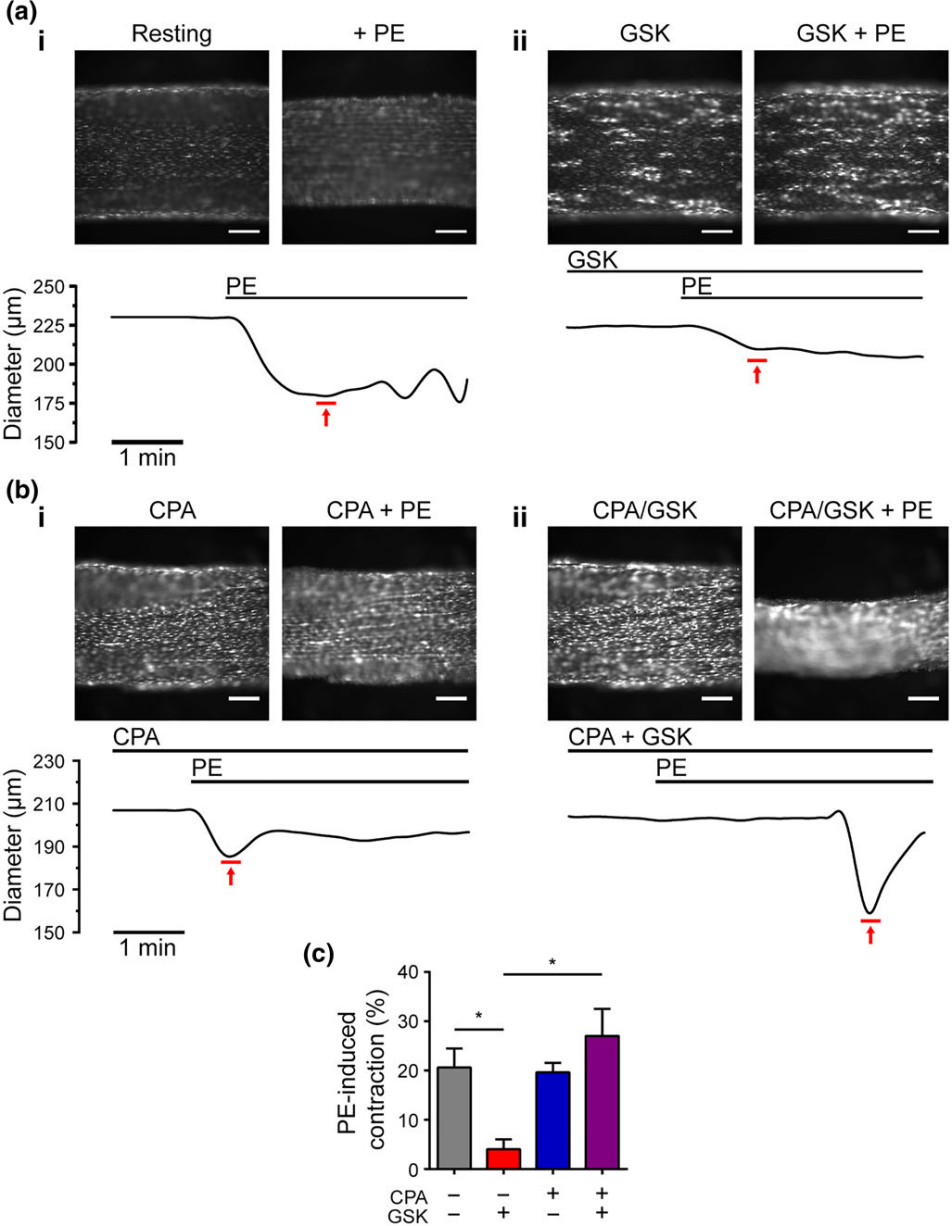
Ca^2+^ store depletion prevents TRPV4‐mediated opposition of vascular tone. (a and b) Effect of GSK1016790A (GSK, 20 nM) on phenylephrine (PE)‐induced contraction in vessels with an intact internal Ca^2+^ store (a) or after depletion of the internal Ca^2+^ store (b) using cyclopiazonic acid (CPA; 6 μm; bottom trace). Upper panels show images of en face arteries, pretreated with the indicated drug, before and after exposure to phenylephrine. Corresponding diameter traces are shown in the lower panels. Data in (i) and (ii) show responses from the same artery. Pretreatment with CPA and GSK consistently delayed the onset but increased the magnitude of the response to phenylephrine. The reason for the delay is not clear. All scale bars = 100 μm. (c) Summary data (mean ± SEM, n = 5) showing the magnitude of phenylephrine‐induced contraction (% resting diameter) under each of the conditions. Diameter measured at the times indicated by the red arrows on diameter traces. *P < .05, significantly different as indicated; repeated measures one‐way ANOVA with multiple comparisons

Depletion of the internal Ca^2+^ store using CPA (6 μM) prevents stable contractions such that it was not possible to reliably assess vasodilator responses in these arteries. Instead, we examined whether pretreatment with GSK was capable of modulating PE‐evoked contraction (Figure 10). Pretreatment with GSK significantly attenuated PE‐induced contractions (Figure 10a, c).

After Ca^2+^ store depletion using CPA, a higher concentration of phenylephrine (3.6 ± 0.2 μM for control; 12.8 ± 2.2 μM for CPA; *n* = 5) was required to achieve a level of contraction (20 ± 2%; *n* = 5) equivalent to control conditions (Figure S2). After store depletion with CPA, pretreatment with GSK no longer attenuated phenylephrine‐induced contraction (4 ± 2% contraction for GSK; 27 ± 6% contraction for GSK + CPA; *n* = 5; *P* < .05). Rather, GSK tended to enhance the contraction to phenylephrine (Figure 10b, c). Together, these results demonstrate that depletion of internal Ca^2+^ stores reduces the inhibitory effect of GSK pretreatment on phenylephrine‐induced contraction.

## 4 DISCUSSION

**Figure 11:**
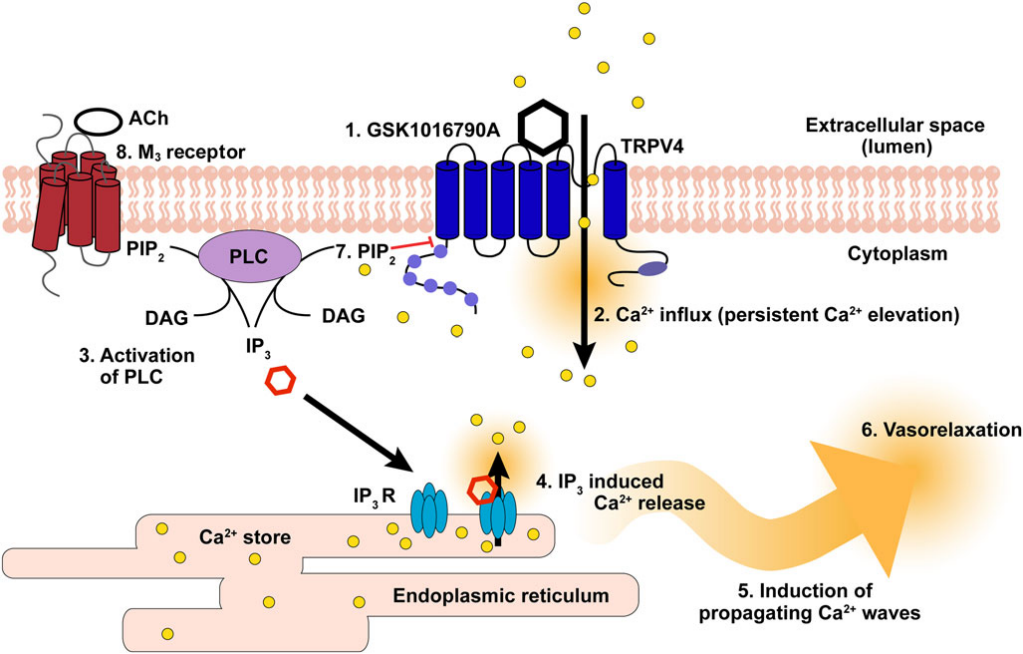
Model of TRPV4‐mediated control of vascular tone. Activation of TRPV4 channels (1) induces Ca^2+^ influx which results in a persistent elevation in cytoplasmic Ca^2+^ concentration (2). Basal (Hardie, Gu, Martin, Sweeney, & Raghu, 2004; Running Deer, Hurley, & Yarfitz, 1995; Willars, Nahorski, & Challiss, 1998) or Ca^2+^‐activated PLC (γ, δ, or β) activity decreases PIP_2_ and generates IP_3_ (3). Ca^2+^ derived from TRPV4 channel activity together with IP_3_ (McCarron, Chalmers, MacMillan, & Olson, 2010) act at IP_3_ receptors on the internal Ca^2+^ store to induce Ca^2+^ release (4). The released Ca^2+^ triggers propagating Ca^2+^ waves (5) by activating neighbouring IP_3_ receptors that ultimately result in vasodilation (6). The entire process remains under the control of Ca^2+^ influx and terminates when TRPV4 channel activity ceases. This channel activity is suppressed by PIP_2_ (7). ACh, via the M3 receptor, also activates PLC to generate IP_3_ and activate Ca^2+^ release from the store. The characteristics of M_3_‐ and TRPV4‐mediated Ca^2+^ release differ significantly (see text)

TRPV4 channels are emerging as key plasmalemmal channels that mediates Ca^2+^ influx and physiological functions in the endothelium (Fiorio Pla et al., 2012; Gao & Wang, 2010; Gifford et al., 2014; Ma et al., 2013; Sayed et al., 2010; Schierling et al., 2011; Sonkusare et al., 2012; Thodeti et al., 2009; Troidl et al., 2009). We have shown that the Ca^2+^ increase in endothelial cells, arising from Ca^2+^ entry via TRPV4 channels, may be amplified by a Ca^2+^‐induced Ca^2+^ release‐like mechanism acting at IP_3_Rs to generate propagating Ca^2+^ waves. When IP_3_R‐mediated Ca^2+^ release was inhibited, TRPV4‐induced increases in intracellular [Ca^2+^] and TRPV4‐mediated control of vascular tone was supressed. These results highlight the significance of endothelial TRPV4‐mediated Ca^2+^‐induced Ca^2+^ release at IP_3_Rs in controlling vascular contractility (Figure 11).

In this study, we analysed individual TRPV4‐mediated Ca^2+^ responses in large numbers of endothelial cells in intact blood vessels. Two components, a slow global increase and propagating Ca^2+^ waves, contributed to the Ca^2+^ response to activation of TRPV4 channels. The response to activation of TRPV4 channels was also heterogeneous among cells. Some cells were highly responsive, while other cells responded weakly or not at all (see also Aird, 2012; Huang, Chu, Chen, & Jen, 2000; Lee et al., 2018; Marie & Beny, 2002; McCarron, Lee, & Wilson, 2017; McCarron et al., 2019; Wilson, et al., 2016). We developed methods to automatically extract and separate the slow global increases and propagating Ca^2+^ waves occurring in each endothelial cell. The changes in Ca^2+^ concentration triggered by activation of TRPV4 channels consisted of different phases which operated sequentially. First, rapid localized Ca^2+^ changes occurred which led to a slow persistent global elevation in Ca^2+^ throughout the cytoplasm. Then, large transient increases as a result of propagating Ca^2+^ waves occurred. The large transient increases as a result of propagating Ca^2+^ waves dominated the Ca^2+^ changes occurring in the endothelium after a short time (~30 s).

Each of the Ca^2+^ changes occurring as a result of activation of TRPV4 channels was critically dependent on Ca^2+^ influx across the plasma membrane. The Ca^2+^ changes were abolished after the removal of external Ca^2+^ or by the inhibition of TRPV4 channels, with RuR or HC067047. These results suggest that Ca^2+^ influx via TRPV4 channels generates the persistent global Ca^2+^ rise. Inhibiting IP_3_‐mediated Ca^2+^ release by depleting the internal Ca^2+^ store, or inhibiting IP_3_Rs, had little effect on the slow persistent global elevation in Ca^2+^ but prevented the large transient increases arising from propagating Ca^2+^ waves. This result suggests that Ca^2+^ entry via TRPV4 channels triggers activation of IP_3_Rs which amplifies the initial Ca^2+^ rise and initiates regenerative release of Ca^2+^ in the form of propagating waves. In support of the active nature of the Ca^2+^ waves, the velocity of wave progression away from the release site was relatively constant within and between cells (~5 to 15 μm·s^−1^). If diffusion alone was responsible for wave progression, velocity and amplitude would decline with distance from the release site. The mechanisms contributing to TRPV4‐mediated activation of Ca^2+^ release and propagation may include direct activation of IP_3_Rs (in a Ca^2+^‐induced Ca^2+^ release like process) or indirect Ca^2+^‐dependent activation of PLC. The partial inhibition of the propagating Ca^2+^ waves by the PLC‐blocker, U73122, is consistent with being a Ca^2+^‐induced Ca^2+^ release‐like process at IP_3_Rs. Basal PLC activity has been reported for many PLC isoforms in biochemical experiments (Running Deer et al., 1995; Willars et al., 1998) and in living cells (Hardie et al., 2004; Willars et al., 1998) which may create a background level of IP_3_ for Ca^2+^‐induced Ca^2+^ release to occur.

Interestingly, blocking of PLC by U73122 also reduced the slow global Ca^2+^ response (i.e., TRPV4‐mediated Ca^2+^ influx). Previous work has shown that TRPV4 channels are inhibited by the phospholipid, phosphatidylinositol 4,5‐bisphosphate (PIP_2_; Harraz, Longden, Hill‐Eubanks, & Nelson, 2018). PLC‐mediated depletion of PIP_2_ results in disinhibition of TRPV4 channels and increased Ca^2+^ influx (Harraz et al., 2018). Thus, blocking of PLC by U73122 might be expected to decrease TRPV4 channel activity.

To block IP_3_Rs, we used both 2‐APB and caffeine. 2‐APB is a broad‐spectrum inhibitor that, although effective in blocking IP_3_Rs in native endothelial cells, may inhibit Ca^2+^ entry pathways (see Peppiatt et al., 2003; Trebak, Bird, McKay, & Putney, 2002; Voets et al., 2001; Wilson, Lee, & McCarron, 2016). However, in the present experimental conditions, 2‐APB did not reduce the slow global Ca^2+^ rise evoked by activation of TRPV4 channels suggesting that it is not inhibiting influx in the present experiments. Caffeine, while often used as a RyR activator, is a potent inhibitor of IP_3_Rs in the same concentration range used to activate RyR (Ehrlich et al., 1994; Parker & Ivorra, 1991; Saleem et al., 2014). Caffeine does not evoke Ca^2+^ release in endothelial cells (Wilson, Saunter, Girkin, & McCarron, 2015) and inhibits Ca^2+^ release induced by photolysis of caged‐IP_3_ in the endothelium (Wilson et al., 2019). That caffeine was effective in inhibiting propagating Ca^2+^ waves supports the conclusion that IP_3_Rs contribute to TRPV4‐mediated Ca^2+^ responses.

RuR and HC067047 were each used to block TRPV4 channels. RuR is an effective TRPV4 channel antagonist but also has effects unrelated to this channnel. For example, RuR is an antagonist of channels such as the mitochondrial uniporter, the RyR, voltage‐dependent Ca^2+^ channels, and other TRP channels. However, the effects of RuR on these other channels are unlikely to explain the present findings. RuR is not membrane permeant and will not have access to the cytoplasm, so the effects on the mitochondrial uniporter or RyR are unlikely to be of significance in the present study. While RuR may have effects on voltage‐sensitive Ca^2+^ channels (Cibulsky & Sather, 1999), these channels do not appear to play a major role in endothelial Ca^2+^ signalling. RuR may also inhibit other TRP channels, for example, TRPC and TRPA. However, those channels are not activated by the agonist used in the present study—GSK1016790A—suggesting that channels other than TRPV4 are unlikely contributors. The present findings with RuR are also supported by the use of the more selective TRPV4 channel antagonist HC067047 which also blocked the response to GSK1016790A. Together, the findings point to TRPV4 channels as being the major target for RuR in the present study.

The increase in cytoplasmic Ca^2+^ concentration evoked by TRPV4‐mediated Ca^2+^‐induced Ca^2+^ release at IP_3_Rs is large and regenerative, as demonstrated by the long distance propagation. This process arises (at least in part) because IP_3_Rs are activated by Ca^2+^. TRPV4 channels are also activated by Ca^2+^ (Strotmann, Schultz, & Plant, 2003). The Ca^2+^‐dependent processes operating at IP_3_Rs and TRPV4 channels are positive feedback mechanisms that should self‐reinforce and reach a maximum in all‐or‐none fashion. However, TRPV4‐mediated Ca^2+^‐induced Ca^2+^ release at IP_3_Rs does not become uncontrolled and, while regenerative, remains tightly regulated by Ca^2+^ influx. The question arises as to why maximum responses do not occur each time TRPV4‐mediated influx begins. The answer may lie in IP_3_Rs and TRPV4 channels being regulated to prevent uncontrolled influx and release (Foskett, White, Cheung, & Mak, 2007; Nilius, Watanabe, & Vriens, 2003). Two observations support this conclusion. First, when TRPV4‐mediated Ca^2+^ influx ceases, the release of Ca^2+^ from IP_3_Rs stops. Thus, Ca^2+^ release is tightly coupled to influx. Second, coordinated activity of IP_3_Rs is required for propagating waves to occur. The propagation of Ca^2+^ waves requires sequential opening and then closing of IP_3_Rs. Thus, after activation, the IP_3_R must become refractory to ensure a directional propagation of the signal. The decline in Ca^2+^ occurring at the back of the wave presumably follows from a functional compartmentalization of the endoplasmic reticulum which renders the site of IP_3_‐mediated Ca^2+^ release refractory to the phosphoinositide. The functional compartmentalization may arise from a lowered local Ca^2+^ concentration in the lumen of the endoplasmic reticulum, or increased Ca^2+^ concentrations could deactivate the receptors (Adkins & Taylor, 1999; McCarron, MacMillan, Bradley, Chalmers, & Muir, 2004; Oancea & Meyer, 1996). These observations highlight a system that is activated by Ca^2+^ influx, but is regulated to prevent an uncontrolled positive feedback processes dominating the TRPV4‐mediated increases in cytoplasmic Ca^2+^ concentrations.

As well as propagating at a constant velocity within cells, TRPV4‐mediated Ca^2+^ waves could move seamlessly, at the same velocity, across cell boundaries into neighbouring cells as intercellular Ca^2+^ waves (Movie S2). These waves were abolished by IP_3_R blockers or removal of GSK. The mechanisms of intercellular wave propagation are not entirely clear, but IP_3_Rs and (since the process stops when GSK is removed) TRPV4 channel activities are both required for these intercellular waves to occur.

In other tissues, intercellular Ca^2+^ waves may be a fundamental mechanism for coordinating multicellular responses (Leybaert & Sanderson, 2012). In the endothelium of small arteries of the mouse cremaster muscle, rapidly spreading intercellular Ca^2+^ waves propagate at a velocity of ∼116 μm·s^−1^ for distances up to ∼1 mm (Tallini et al., 2007). These intercellular waves are associated with vasodilation and are hypothesized to regulate blood flow to the parenchyma by inducing upstream dilation of arterioles (Tallini et al., 2007). The intercellular waves observed in the present study had a slower velocity (~5 to 15 μm·s^−1^) than those in mouse cremaster arterioles, suggesting a different physiological control mechanism.

In previous studies, when endothelial IP_3_R‐mediated signalling was eliminated by depletion of the internal Ca^2+^ store, activation of TRPV4 channels induced a large but highly localized (few square micrometres) increase in Ca^2+^ concentrations (Sonkusare et al., 2012). These localized Ca^2+^ signals were reported to activate endothelial Ca^2+^‐activated K^+^ channels (Sonkusare et al., 2012) and lead to endothelial hyperpolarization and vasodilation (Sonkusare et al., 2012). However, activation of TRPV4 channels is known to also control the orientation of endothelial cells, regulate endothelial permeability, and modulate the production of antithrombotic factors, each of which may require a more global [Ca^2+^] increase throughout the cytoplasm (Noren et al., 2016; Phuong et al., 2017; Thodeti et al., 2009; Thoppil et al., 2016). In the present study, IP_3_R‐mediated Ca^2+^ release generated propagating Ca^2+^ waves and provided a mechanism by which TRPV4 channel activity, in the presence of a functioning Ca^2+^ store, may generate large rises in Ca^2+^ throughout the cytoplasm. These propagating waves are critical for TRPV4‐mediated control of vascular tone. When Ca^2+^ release from the internal store is inhibited, TRPV4‐mediated endothelial control of tone is abolished (Figure 10). This result suggests that control of vascular tone by TRPV4 channels is mediated by Ca^2+^‐induced Ca^2+^ release via IP_3_Rs. It is tempting to also speculate that the more general rise in Ca^2+^ throughout the cells, which occurs as a result of the propagating IP_3_R mediated Ca^2+^ waves, will facilitate TRPV4‐mediated control of endothelial permeability and the production of antithrombotic factors (Noren et al., 2016; Phuong et al., 2017; Thodeti et al., 2009; Thoppil et al., 2016).

ACh evoked rapid asynchronous Ca^2+^ waves in neighbouring endothelial cells. ACh‐evoked changes in intracellular Ca^2+^ concentration are linked closely to the IP_3_‐sensitive Ca^2+^ store, but they do not involve TRPV4 channels in rat arteries (Hartmannsgruber et al., 2007; see also Kohler et al., 2006; Wilson, Lee, & McCarron, 2016; Figure 10). While the response to the activation by ACh and activation of TRPV4 channels each involved IP_3_, the Ca^2+^ signals generated had very different characteristics. ACh‐evoked Ca^2+^ increases did not appear to coordinate between cells. Activation of TRPV4 channels evoked a slowly increasing baseline and large propagating Ca^2+^ waves.

Endothelial TRPV4 channels are involved in several cardiovascular control mechanisms, including inhibiting vasoconstriction in small skeletal muscle arteries in response to increases in temperature (Gifford et al., 2014), regulating myogenic tone (Bagher et al., 2012), remodelling of the cytoskeleton and reorientation of the endothelial cells in response to mechanical forces (Thodeti et al., 2009), collateral vessel growth (Sayed et al., 2010; Schierling et al., 2011; Troidl et al., 2009), and arachidonic acid‐induced endothelial cell migration required for angiogenesis (Fiorio Pla et al., 2012). The present results show that when the internal IP_3_ sensitive store is intact, TRPV4 channel activity evokes IP_3_R signalling to generate Ca^2+^ waves that propagate within and between cells, and through the Ca^2+^ store, TRPV4 channels modulate vascular contractility. The results demonstrate a link between TRPV4 channel activity and Ca^2+^‐induced Ca^2+^ release at IP_3_R in endothelial cells and offer a new target for drug development.

## CONFLICT OF INTEREST

The authors declare no conflicts of interest.

## AUTHOR CONTRIBUTIONS

H.R.H., M.D.L.,C.W., and J.G.M. developed the concept. H.R.H., M.D.L., X.Z., and C.W. performed the experiments. C.W. and C.D.S. wrote the analysis software. H.R.H., M.D.L., X.Z., and C.W. analysed the data. J.G.M. and H.R.H. drafted the manuscript. J.G.M., H.R.H., C.W., C.D.S., and M.D.L. edited the manuscript. J.G.M., M.D.L., C.W., and X.Z. revised the manuscript. C.W., C.D.S., and J.G.M. sourced funding. All authors approved the final version of the manuscript.

## DECLARATION OF TRANSPARENCY AND SCIENTIFIC RIGOUR

This Declaration acknowledges that this paper adheres to the principles for transparent reporting and scientific rigour of preclinical research as stated in the *BJP* guidelines for Design & Analysis and Animal Experimentation, and as recommended by funding agencies, publishers and other organisations engaged with supporting research

## DATA AVAILABILITY

All data underpinning this study is available from the authors upon reasonable request.

## Supporting information

Data S1: List of IUPHAR Hyperlinks

Figure S1: Extraction of Ca2+ signals from raw data. A) Ca2+ image displaying a field of 97 native mesenteric artery endothelial cells. The centre of each cell is marked with a red dot. B) F/F0 Ca2+ traces for each of the cells identified in (A). The baseline region (red) was automatically identified for each cell, a trace from a single cell is highlighted in bold. C‐E) To extract signalling metrics, fast and slow Ca2+ signal components were extracted from F/F0 traces. C) Example of Ca2+ signal de‐multiplexing for the cellular trace (#57) highlighted in B. The time‐dependent baseline (slow component, blue) of the F/F0 signal (black) was calculated using an asymmetric least‐squares (ALS) function, and modelled by a sigmoid function (green). The fast component of the signal (red) was isolated by dividing the F/F0 signal by the ALS signal. D) Sigmoid function, used to extract the rise time of the slow signal component, and fast signal component (shown in C) shown on an expanded time scale. Peaks were identified in the fast signal using an automated peak detection algorithm. E) Original F/F0, de‐multiplexed signal components, and sigmoid fits for each of the signals shown in B. Each trace is coloured according to the magnitude of the ALS signal, mean signal is overlaid in black.
Figure S2: ACh and GSK dilate PE‐constricted mesenteric arteries. A) Mean PE‐contracted vessel diameter (% of resting, left) and corresponding concentration of PE (right), before (+ endothelium) and after (‐ endothelium) mechanical removal of the endothelium (n = 10). B) Mean PE‐contracted vessel diameter (% of resting, left) and corresponding concentration of PE (right), in the absence (‐) and presence (+) of cyclopiazonic acid (CPA; 6 μM; n = 5). C) Summary data showing the effect of ACh (blue) and GSK (red) on the diameter of PE‐constricted arteries. Statistical analyses were performed using paired Student's t‐test (A), unpaired Student's t‐test (B) and repeated measures one‐way ANOVA with multiple comparisons (C). * = p < 0.05.
Figure S3: Repeat application of GSK elicits reproducible Ca2+ signals. A) Schematic of the experimental protocol. Mesenteric artery endothelial cells were subjected to perfusion with GSK1016790A (GSK; 20 nM) prior to washing, and a second application of GSK (20 nM). B) Composite images showing basal Cal‐520/AM fluorescence (grey) with Ca2+ activity overlaid (red). Ca2+ activity was derived from the 120 sec periods indicated by the numbered arrows in A. Scale bars = 50 μm. C) GSK‐evoked Ca2+ signals for the 1st (left) and 2nd (right) application of GSK. D) GSK‐evoked Ca2+ signalling parameters. Upper panels show the mean response for individual cells (grey dots) for a single experiment. The experimental mean is overlaid in red or blue. Lower panel show paired summary data (grand mean) for all experimental replicates. Parameters extracted include: the percentage of cells activated, the mean ΔF/F0 of Ca2+ peaks, the number of peaks detected per cell (peaks/cell), the amplitude of the fitted sigmoid, as well as the duration, rise time, fall time, area under the curve (AUC) of all Ca2+ signal peaks. * p < 0.05 (n=13) using paired Student's t‐test.
Figure S4: TRPV4‐mediated local Ca2+ activity. A) Local Ca2+ events elicited by GSK101679A (GSK; 20 nM). Panels show a ΔF/F0 time series of a single endothelial cell. Ca2+ events initiate at, but remain localized within, either end of the cell. Scale bars = 20 μm. B) Ca2+ traces from the entire cell (red) or from circular ROIs (~2.5 μm diameter) positioned at each of the Ca2+ event initiation sites. Local Ca2+ events are inconsistently reflected in global (whole‐cell) Ca2+ traces.
Figure S5: Acetylcholine and GSK101679A stimulate different patterns of signalling across multiple cells. Left; Image showing the basal Cal‐520/AM fluorescence (grey) in a field of mesenteric artery endothelial cells. Scale bar = 50 μm. A transect (x‐y) was drawn through three adjoining cells (1‐3). Right) Ca2+ signals (F/F0) and kymographs illustrating Cal‐520/AM fluorescence (F) along the transect (x‐y; vertical axis) for cells 1‐3, when stimulated with acetylcholine (ACh; 100 nM; upper right) and then, after washout and recovery, GSK101679A (GSK; 20 nM; lower right). Traces are coloured according to the assigned cell number and kymographs are coloured on a scale where dark blue = min F and dark red = max F.
Figure S6: U73343, the inactive analogue of U73122, did not alter GSK induced Ca2+ signals. A) Composite images showing GSK1016790A (GSK)‐evoked Ca2+ activity in the absence (left) and presence (right) of U73343 (2 μM; 10 mins), the inactive analogue of the PLC inhibitor U73122 (Figure 6). Images show basal Cal‐520/AM fluorescence (grey) with Ca2+ activity overlaid (red). Scale bars = 50 μm. B) GSK‐evoked (20 nM; grey box) Ca2+ signals (F/F0; top) were decomposed into propagating Ca2+ waves (fast; middle) and slow global Ca2+ rise (slow; bottom) components. C) Summary data illustrating the percentage of cells displaying propagating Ca2+ waves and slow global Ca2+ rises. D‐F) Paired summary data showing the effect of U73343 on Ca2+ wave propagation velocity (D), peak amplitude (ΔF/F0, E) and the number of peaks per cell (F). The left plots in E‐F are scatter plots showing the mean Ca2+ event amplitude, or oscillation frequency, within each cell visualised in the experiment shown in panels A‐B. Individual data points are coloured (from blue, low to red, high) according to the density (i.e. occurrence) of particular values. * p < 0.05 using paired Student's t‐test (n = 5).
Figure S7: U73122 does not alter IP3‐mediated Ca2+ signals. A) Composite image (max F/F0 intensity projection) illustrating the Ca2+ response to photolysis of caged‐IP3 in the absence (upper) and presence (lower) of U73122 (2 μM, 10 mins). B) Whole‐cell Ca2+ traces extracted from the data shown in A. C) Summary data showing the effect of U73122 on the peak amplitude and number of cells that responded to photorelease of caged IP3. Each data point indicates the mean from a single field of endothelial cells (one animal). *, p < 0.05; NS, no statistically significant difference detected (i.e. p > 0.05) using paired t test. Scale bars = 10 μm.
Figure S8: Caffeine inhibits propagating Ca2+ waves induced by TRPV4 activation. A) Composite images showing GSK1016790A (GSK)‐evoked Ca2+ activity in native mesenteric artery endothelial cells in the absence (left) and presence of caffeine (10 mM; right). Images show basal Cal‐520/AM fluorescence (grey) with Ca2+ activity overlaid (red). Scale bars = 50 μm. B) Raw F/F0 (top), propagating Ca2+ waves (fast; middle), and slow Ca2+ rises (slow; bottom) GSK‐evoked (20 nM; grey box) Ca2+ signal components in the absence (left) and presence (right) of caffeine. C) Summary data illustrating the percentage of cells exhibiting fast and slow signal components. D‐F) Paired summary data showing the effect of IP3R inhibition on Ca2+ wave propagation velocity (D), peak amplitude (ΔF/F0, E) and the number of peaks per cell (F). The left plots in E‐F are scatter plots showing the mean Ca2+ event amplitude, or oscillation frequency, within each cell visualised in the experiment shown in panels A‐B. Individual data points are coloured (from blue, low to red, high) according to the density (i.e. occurrence) of particular values. * p < 0.05 using paired Student's t‐test (n = 5).
Figure S9: GSK‐evoked Ca2+ signalling returns towards basal levels during a washout period. Ca2+ signals (F/F0; top) extracted from mesenteric artery endothelial cells stimulated with GSK1016790A (GSK; 20 nM). When GSK was washed out, signals returned to resting levels. Composite images (bottom) showing basal Cal‐520/AM fluorescence (grey) with Ca2+ activity overlaid (red). Ca2+ activity was derived from the 60 sec periods indicated by the dashed lines. Scale bars = 50 μm.
Figure S10: Acetylcholine (ACh) evoked relaxation is independent of TRPV4 in rat mesenteric arteries. (A) Representative diameter trace of a second order mesenteric artery (60 mmHg; luminal flow rate ~100 μl/min) in response to ACh or GSK. Upon exposure to PE (~500 nM; duration indicated by the bar above the trace), vessels contracted and underwent vasomotion. Upon subsequent exposure to ACh (100 nM), arteries relaxed back towards resting diameter. On wash‐out of ACh, arteries contracted again. GSK (20 nM) also relaxed arteries back towards resting diameter. Dilation to GSK was reversed by the TRPV4 antagonist, HC067047 (10 μM). However, HC067047 did not prevent dilation to ACh. (B) Example images from the artery in A showing the artery prior to PE exposure (basal), after PE, after ACh, after GSK, after GSK and HC067047, and after ACh with HC067047. The red horizontal lines are measurement scan lines created by Vasotracker (see Methods). (C) Summary data showing the percentage relaxation to ACh and GSK1016790A (GSK) in the absence (black bar) and present (open bar) of HC067047 (n=5; * p<0.05).

Movie S1: Endothelial Ca2+ signalling evoked by GSK. The grey channel shows basal Ca2+ levels,while the red channel shows Ca2+ activity (red, determined by sequential subtraction) evoked by the TRPV4 agonist, GSK1016790A. Data acquired at 10 Hz. Scale bar = 20 μm.

Movie S2: GSK‐evoked multicellular Ca2+ waves. Pseudo‐coloured active Ca2+ wave fronts (determined by sequential subtraction) showing a GSK‐evoked (20 nM) Ca2+ wave that propagates across four endothelial cells. Cell outlines are overlaid in white. Data acquired at 20 Hz. Scale bar = 20 μm.

Movie S3: Ca2+ waves stop upon washout of GSK. The grey channel shows basal Ca2+ levels,while the red channel shows Ca2+ activity (red, determined by sequential subtraction). GSK (20 nM) present from start of video and washed out when indicated. Data acquired at 10 Hz. Scale bar = 20 μm.

## References

[bph14762-bib-0001] Adkins, C. E. , & Taylor, C. W. (1999). Lateral inhibition of inositol 1,4,5‐trisphosphate receptors by cytosolic Ca^2+^ . Current Biology, 9, 1115–1118. 10.1016/S0960-9822(99)80481-3 10531009

[bph14762-bib-0002] Aird, W. C. (2012). Endothelial cell heterogeneity. Cold Spring Harbor Perspectives in Medicine, 2(1), a006429 10.1101/cshperspect.a006429 22315715 PMC3253027

[bph14762-bib-0003] Alexander, S. P. H. , Christopoulos, A. , Davenport, A. P. , Kelly, E. , Marrion, N. V. , Peters, J. A. , … CGTP Collaborators . (2017). The Concise Guide to PHARMACOLOGY 2017/18: G protein‐coupled receptors. British Journal of Pharmacology, 174, S17–S129. 10.1111/bph.13878 29055040 PMC5650667

[bph14762-bib-0004] Alexander, S. P. H. , Fabbro, D. , Kelly, E. , Marrion, N. V. , Peters, J. A. , Faccenda, E. , … CGTP Collaborators . (2017). The Concise Guide to PHARMACOLOGY 2017/18: Enzymes. British Journal of Pharmacology, 174, S272–S359. 10.1111/bph.13877 29055034 PMC5650666

[bph14762-bib-0005] Alexander, S. P. H. , Peters, J. A. , Kelly, E. , Marrion, N. V. , Faccenda, E. , Harding, S. D. , … CGTP Collaborators . (2017). The Concise Guide to PHARMACOLOGY 2017/18: Ligand‐gated ion channels. British Journal of Pharmacology, 174, S130–S159. 10.1111/bph.13879 29055038 PMC5650660

[bph14762-bib-0006] Alexander, S. P. H. , Striessnig, J. , Kelly, E. , Marrion, N. V. , Peters, J. A. , Faccenda, E. , … CGTP Collaborators . (2017). The Concise Guide to PHARMACOLOGY 2017/18: Voltage‐gated ion channels. British Journal of Pharmacology, 174, S160–S194. 10.1111/bph.13884 29055033 PMC5650668

[bph14762-bib-0007] Bagher, P. , Beleznai, T. , Kansui, Y. , Mitchell, R. , Garland, C. J. , & Dora, K. A. (2012). Low intravascular pressure activates endothelial cell TRPV4 channels, local Ca^2+^ events, and IKCa channels, reducing arteriolar tone. Proceedings of the National Academy of Sciences of the United States of America, 109(44), 18174–18179. 10.1073/pnas.1211946109 23071308 PMC3497745

[bph14762-bib-0008] Berridge, M. J. (1997). Elementary and global aspects of calcium signalling. The Journal of Experimental Biology, 200(Pt 2), 315–319. 9050239 10.1242/jeb.200.2.315

[bph14762-bib-0009] Bootman, M. , Niggli, E. , Berridge, M. , & Lipp, P. (1997). Imaging the hierarchical Ca^2+^ signalling system in HeLa cells. The Journal of Physiology, 499, 307–314. 10.1113/jphysiol.1997.sp021928 9080361 PMC1159306

[bph14762-bib-0010] Bootman, M. D. , Berridge, M. J. , & Lipp, P. (1997). Cooking with calcium: The recipes for composing global signals from elementary events. Cell, 91, 367–373. 10.1016/S0092-8674(00)80420-1 9363945

[bph14762-bib-0011] Borisova, L. , Wray, S. , Eisner, D. A. , & Burdyga, T. (2009). How structure, Ca signals, and cellular communications underlie function in precapillary arterioles. Circulation Research, 105(8), 803–810. 10.1161/CIRCRESAHA.109.202960 19713534

[bph14762-bib-0012] Catterall, W. A. (2011). Voltage‐gated calcium channels. Cold Spring Harbor Perspectives in Biology, 3(8), a003947 10.1101/cshperspect.a003947 21746798 PMC3140680

[bph14762-bib-0013] Cibulsky, S. M. , & Sather, W. A. (1999). Block by ruthenium red of cloned neuronal voltage‐gated calcium channels. The Journal of Pharmacology and Experimental Therapeutics, 289(3), 1447–1453. 10336538

[bph14762-bib-0014] Dunn, K. M. , Hill‐Eubanks, D. C. , Liedtke, W. B. , & Nelson, M. T. (2013). TRPV4 channels stimulate Ca^2+^‐induced Ca^2+^ release in astrocytic endfeet and amplify neurovascular coupling responses. Proceedings of the National Academy of Sciences of the United States of America, 110(15), 6157–6162. 10.1073/pnas.1216514110 23530219 PMC3625327

[bph14762-bib-0015] Earley, S. , Heppner, T. J. , Nelson, M. T. , & Brayden, J. E. (2005). TRPV4 forms a novel Ca^2+^ signaling complex with ryanodine receptors and BKCa channels. Circulation Research, 97(12), 1270–1279. 10.1161/01.RES.0000194321.60300.d6 16269659

[bph14762-bib-0016] Ehrlich, B. E. , Kaftan, E. , Bezprozvannaya, S. , & Bezprozvanny, I. (1994). The pharmacology of intracellular Ca^2+^‐release channels. Trends in Pharmacological Sciences, 15(5), 145–149. 10.1016/0165-6147(94)90074-4 7754532

[bph14762-bib-0017] Eilers, P. H. C. , & Boelens, H. F. M. (2005). Baseline correction with asymmetric least squares smoothing Leiden University Medical Centre Report.

[bph14762-bib-0018] Filosa, J. A. , Yao, X. , & Rath, G. (2013). TRPV4 and the regulation of vascular tone. Journal of Cardiovascular Pharmacology, 61(2), 113–119. 10.1097/FJC.0b013e318279ba42 23107877 PMC3564998

[bph14762-bib-0019] Fiorio Pla, A. , Ong, H. L. , Cheng, K. T. , Brossa, A. , Bussolati, B. , Lockwich, T. , … Ambudkar, I. S. (2012). TRPV4 mediates tumor‐derived endothelial cell migration via arachidonic acid‐activated actin remodeling. Oncogene, 31(2), 200–212. 10.1038/onc.2011.231 21685934 PMC5934994

[bph14762-bib-0020] Foskett, J. K. , White, C. , Cheung, K. H. , & Mak, D. O. (2007). Inositol trisphosphate receptor Ca^2+^ release channels. Physiological Reviews, 87(2), 593–658. 10.1152/physrev.00035.2006 17429043 PMC2901638

[bph14762-bib-0021] Freichel, M. , Suh, S. H. , Pfeifer, A. , Schweig, U. , Trost, C. , Weissgerber, P. , … Nilius, B. (2001). Lack of an endothelial store‐operated Ca^2+^ current impairs agonist‐dependent vasorelaxation in TRP4^−/−^ mice. Nature Cell Biology, 3(2), 121–127. 10.1038/35055019 11175743

[bph14762-bib-0022] Gao, F. , & Wang, D. H. (2010). Hypotension induced by activation of the transient receptor potential vanilloid 4 channels: Role of Ca^2+^‐activated K^+^ channels and sensory nerves. Journal of Hypertension, 28(1), 102–110. 10.1097/HJH.0b013e328332b865 19996988 PMC3515639

[bph14762-bib-0023] Gao, X. , Wu, L. , & O'Neil, R. G. (2003). Temperature‐modulated diversity of TRPV4 channel gating: Activation by physical stresses and phorbol ester derivatives through protein kinase C‐dependent and ‐independent pathways. The Journal of Biological Chemistry, 278(29), 27129–27137. 10.1074/jbc.M302517200 12738791

[bph14762-bib-0024] Gifford, J. R. , Ives, S. J. , Park, S. Y. , Andtbacka, R. H. , Hyngstrom, J. R. , Mueller, M. T. , … Richardson, R. S. (2014). α1‐ and α2‐adrenergic responsiveness in human skeletal muscle feed arteries: The role of TRPV ion channels in heat‐induced sympatholysis. American Journal of Physiology. Heart and Circulatory Physiology, 307(9), H1288–H1297. 10.1152/ajpheart.00068.2014 25172894 PMC4217010

[bph14762-bib-0025] Hardie, R. C. , Gu, Y. , Martin, F. , Sweeney, S. T. , & Raghu, P. (2004). In vivo light‐induced and basal phospholipase C activity in Drosophila photoreceptors measured with genetically targeted phosphatidylinositol 4,5‐bisphosphate‐sensitive ion channels (Kir2.1). The Journal of Biological Chemistry, 279(46), 47773–47782. 10.1074/jbc.M407525200 15355960

[bph14762-bib-0026] Harding, S. D. , Sharman, J. L. , Faccenda, E. , Southan, C. , Pawson, A. J. , Ireland, S. , … NC‐IUPHAR . (2018). The IUPHAR/BPS guide to pharmacology in 2018: Updates and expansion to encompass the new guide to immunopharmacology. Nucleic Acids Research, 46(D1), D1091–D1106. 10.1093/nar/gkx1121 29149325 PMC5753190

[bph14762-bib-0027] Harraz, O. F. , Longden, T. A. , Hill‐Eubanks, D. , & Nelson, M. T. (2018). PIP2 depletion promotes TRPV4 channel activity in mouse brain capillary endothelial cells. eLife, 7 10.7554/eLife.38689 PMC6117155 30084828

[bph14762-bib-0028] Hartmannsgruber, V. , Heyken, W. T. , Kacik, M. , Kaistha, A. , Grgic, I. , Harteneck, C. , … Köhler, R. (2007). Arterial response to shear stress critically depends on endothelial TRPV4 expression. PLoS ONE, 2(9), e827 10.1371/journal.pone.0000827 17786199 PMC1959246

[bph14762-bib-0029] Hofmann, T. , Schaefer, M. , Schultz, G. , & Gudermann, T. (2002). Subunit composition of mammalian transient receptor potential channels in living cells. Proceedings of the National Academy of Sciences of the United States of America, 99(11), 7461–7466. 10.1073/pnas.102596199 12032305 PMC124253

[bph14762-bib-0030] Huang, T. Y. , Chu, T. F. , Chen, H. I. , & Jen, C. J. (2000). Heterogeneity of [Ca^2+^]_i_ signaling in intact rat aortic endothelium. The FASEB Journal, 14(5), 797–804. 10.1096/fasebj.14.5.797 10744636

[bph14762-bib-0031] Kohler, R. , Heyken, W. T. , Heinau, P. , Schubert, R. , Si, H. , Kacik, M. , … Hoyer, J. (2006). Evidence for a functional role of endothelial transient receptor potential V4 in shear stress‐induced vasodilatation. Arteriosclerosis, Thrombosis, and Vascular Biology, 26(7), 1495–1502. 10.1161/01.ATV.0000225698.36212.6a 16675722

[bph14762-bib-0032] Kilkenny, C. , Browne, W. , Cuthill, I. C. , Emerson, M. , & Altman, D. G. (2010). Animal research: Reporting *in vivo* experiments: The ARRIVE guidelines. British Journal of Pharmacology, 160, 1577–1579. 20649561 10.1111/j.1476-5381.2010.00872.x PMC2936830

[bph14762-bib-0033] Lawton, P. F. , Lee, M. D. , Saunter, C. D. , Girkin, J. M. , McCarron, J. G. , & Wilson, C. (2019). VasoTracker, a low‐cost and open source pressure myograph system for vascular physiology has been approved for production and accepted for publication in Frontiers in Physiology, section Vascular Physiology. Frontiers in Physiology, 10 10.3389/fphys.2019.00099 PMC6393368 30846942

[bph14762-bib-0034] Lee, M. D. , Wilson, C. , Saunter, C. D. , Kennedy, C. , Girkin, J. M. , & McCarron, J. G. (2018). Spatially structured cell populations process multiple sensory signals in parallel in intact vascular endothelium. Science Signaling, 11(561), eaar4411 10.1126/scisignal.aar4411 30563865 PMC6420068

[bph14762-bib-0035] Leybaert, L. , & Sanderson, M. J. (2012). Intercellular Ca^2+^ waves: Mechanisms and function. Physiological Reviews, 92(3), 1359–1392. 10.1152/physrev.00029.2011 22811430 PMC4496049

[bph14762-bib-0036] Liedtke, W. , Choe, Y. , Marti‐Renom, M. A. , Bell, A. M. , Denis, C. S. , Sali, A. , … Heller, S. (2000). Vanilloid receptor‐related osmotically activated channel (VR‐OAC), a candidate vertebrate osmoreceptor. Cell, 103(3), 525–535. 10.1016/S0092-8674(00)00143-4 11081638 PMC2211528

[bph14762-bib-0037] Liedtke, W. , Tobin, D. M. , Bargmann, C. I. , & Friedman, J. M. (2003). Mammalian TRPV4 (VR‐OAC) directs behavioral responses to osmotic and mechanical stimuli in *Caenorhabditis elegans* . Proceedings of the National Academy of Sciences of the United States of America, 100(Suppl 2), 14531–14536. 10.1073/pnas.2235619100 14581619 PMC304114

[bph14762-bib-0038] Ma, X. , Du, J. , Zhang, P. , Deng, J. , Liu, J. , Lam, F. F. , … Yao, X. (2013). Functional role of TRPV4‐KCa2.3 signaling in vascular endothelial cells in normal and streptozotocin‐induced diabetic rats. Hypertension, 62(1), 134–139. 10.1161/HYPERTENSIONAHA.113.01500 23648706

[bph14762-bib-0039] Macmillan, D. , & McCarron, J. G. (2010). The phospholipase C inhibitor U‐73122 inhibits Ca^2+^ release from the intracellular sarcoplasmic reticulum Ca^2+^ store by inhibiting Ca^2+^ pumps in smooth muscle. British Journal of Pharmacology, 160(6), 1295–1301. 10.1111/j.1476-5381.2010.00771.x 20590621 PMC2938802

[bph14762-bib-0040] Mannaa, M. , Marko, L. , Balogh, A. , Vigolo, E. , N'Diaye, G. , Kassmann, M. , … Gollasch, M. (2018). Transient receptor potential vanilloid 4 channel deficiency aggravates tubular damage after acute renal ischaemia reperfusion. Scientific Reports, 8(1), 4878 10.1038/s41598-018-23165-0 29559678 PMC5861116

[bph14762-bib-0041] Marie, I. , & Beny, J. L. (2002). Calcium imaging of murine thoracic aorta endothelium by confocal microscopy reveals inhomogeneous distribution of endothelial cells responding to vasodilator agents. Journal of Vascular Research, 39(3), 260–267. 10.1159/000063691 12097824

[bph14762-bib-0042] Marrelli, S. P. , O'Neil, R. G. , Brown, R. C. , & Bryan, R. M. Jr. (2007). PLA2 and TRPV4 channels regulate endothelial calcium in cerebral arteries. American Journal of Physiology. Heart and Circulatory Physiology, 292(3), H1390–H1397. 10.1152/ajpheart.01006.2006 17071727

[bph14762-bib-0043] McCarron, J. G. , Chalmers, S. , Bradley, K. N. , Macmillan, D. , & Muir, T. C. (2006). Ca^2+^ microdomains in smooth muscle. Cell Calcium, 40, 461–493. 10.1016/j.ceca.2006.08.010 17069885

[bph14762-bib-0044] McCarron, J. G. , Chalmers, S. , MacMillan, D. , & Olson, M. L. (2010). Agonist‐evoked Ca^2+^ wave progression requires Ca^2+^ and IP_3_ . Journal of Cell Physiology, 244, 334–344. 10.1002/jcp.22103 PMC3947531 20432430

[bph14762-bib-0045] McCarron, J. G. , Lee, M. D. , & Wilson, C. (2017). The endothelium solves problems that endothelial cells do not know exist. Trends in Pharmacological Sciences, 38(4), 322–338. 10.1016/j.tips.2017.01.008 28214012 PMC5381697

[bph14762-bib-0046] McCarron, J. G. , MacMillan, D. , Bradley, K. N. , Chalmers, S. , & Muir, T. C. (2004). Origin and mechanisms of Ca^2+^ waves in smooth muscle as revealed by localized photolysis of caged inositol 1,4,5‐trisphosphate. The Journal of Biological Chemistry, 279, 8417–8427. 10.1074/jbc.M311797200 14660609

[bph14762-bib-0047] McCarron, J. G. , Wilson, C. , Heathcote, H. R. , Zhang, X. , Buckley, C. , & Lee, M. D. (2019). Heterogeneity and emergent behaviour in the vascular endothelium. Current Opinion in Pharmacology, 45, 23–32. 10.1016/j.coph.2019.03.008 31005824 PMC6700393

[bph14762-bib-0048] Mendoza, S. A. , Fang, J. , Gutterman, D. D. , Wilcox, D. A. , Bubolz, A. H. , Li, R. , … Zhang, D. X. (2010). TRPV4‐mediated endothelial Ca^2+^ influx and vasodilation in response to shear stress. American Journal of Physiology. Heart and Circulatory Physiology, 298(2), H466–H476. 10.1152/ajpheart.00854.2009 19966050 PMC2822567

[bph14762-bib-0049] Mizuno, A. , Matsumoto, N. , Imai, M. , & Suzuki, M. (2003). Impaired osmotic sensation in mice lacking TRPV4. American Journal of Physiology. Cell Physiology, 285(1), C96–C101. 10.1152/ajpcell.00559.2002 12777254

[bph14762-bib-0050] Moccia, F. , Berra‐Romani, R. , & Tanzi, F. (2012). Update on vascular endothelial Ca^2+^ signalling: A tale of ion channels, pumps and transporters. World Journal of Biological Chemistry, 3(7), 127–158. 10.4331/wjbc.v3.i7.127 22905291 PMC3421132

[bph14762-bib-0051] Moore, C. , Cevikbas, F. , Pasolli, H. A. , Chen, Y. , Kong, W. , Kempkes, C. , … Liedtke, W. B. (2013). UVB radiation generates sunburn pain and affects skin by activating epidermal TRPV4 ion channels and triggering endothelin‐1 signaling. Proceedings of the National Academy of Sciences of the United States of America, 110(34), E3225–E3234. 10.1073/pnas.1312933110 23929777 PMC3752269

[bph14762-bib-0052] Mumtaz, S. , Burdyga, G. , Borisova, L. , Wray, S. , & Burdyga, T. (2011). The mechanism of agonist induced Ca^2+^ signalling in intact endothelial cells studied confocally in in situ arteries. Cell Calcium, 49(1), 66–77. 10.1016/j.ceca.2010.11.010 21176847 PMC3098389

[bph14762-bib-0053] Nelson, M. T. , Patlak, J. B. , Worley, J. F. , & Standen, N. B. (1990). Calcium channels, potassium channels, and voltage dependence of arterial smooth muscle tone. The American Journal of Physiology, 259(1 Pt 1), C3–C18. 10.1152/ajpcell.1990.259.1.C3 2164782

[bph14762-bib-0054] Nilius, B. , Droogmans, G. , & Wondergem, R. (2003). Transient receptor potential channels in endothelium: Solving the calcium entry puzzle? Endothelium: Journal of Endothelial Cell Research, 10(1), 5–15. 10.1080/10623320303356 12699072

[bph14762-bib-0055] Nilius, B. , & Voets, T. (2013). The puzzle of TRPV4 channelopathies. EMBO Reports, 14(2), 152–163. 10.1038/embor.2012.219 23306656 PMC3566843

[bph14762-bib-0056] Nilius, B. , Watanabe, H. , & Vriens, J. (2003). The TRPV4 channel: Structure–function relationship and promiscuous gating behaviour. Pflügers Archiv, 446(3), 298–303. 10.1007/s00424-003-1028-9 12715179

[bph14762-bib-0057] Noren, D. P. , Chou, W. H. , Lee, S. H. , Qutub, A. A. , Warmflash, A. , Wagner, D. S. , … Levchenko, A. (2016). Endothelial cells decode VEGF‐mediated Ca^2+^ signaling patterns to produce distinct functional responses. Science Signaling, 9(416), ra20 10.1126/scisignal.aad3188 26905425 PMC5301990

[bph14762-bib-0058] Oancea, E. , & Meyer, T. (1996). Reversible desensitization of inositol trisphosphate‐induced calcium release provides a mechanism for repetitive calcium spikes. The Journal of Biological Chemistry, 271, 17253–17260. 10.1074/jbc.271.29.17253 8663416

[bph14762-bib-0059] Olson, M. L. , Chalmers, S. , & McCarron, J. G. (2012). Mitochondrial organization and Ca^2+^ uptake. Biochemical Society Transactions, 40(1), 158–167. 10.1042/BST20110705 22260683

[bph14762-bib-0060] Olson, M. L. , Sandison, M. E. , Chalmers, S. , & McCarron, J. G. (2012). Microdomains of muscarinic acetylcholine and Ins(1,4,5)P_3_ receptors create ‘Ins(1,4,5)P_3_ junctions’ and sites of Ca^2+^ wave initiation in smooth muscle. Journal of Cell Science, 125(Pt 22, 5315–5328. 10.1242/jcs.105163 22946060 PMC3561854

[bph14762-bib-0061] Parker, I. , & Ivorra, I. (1991). Caffeine inhibits inositol trisphosphate‐mediated liberation of intracellular calcium in Xenopus oocytes. The Journal of Physiology, 433, 229–240. 10.1113/jphysiol.1991.sp018423 1844813 PMC1181368

[bph14762-bib-0062] Peppiatt, C. M. , Collins, T. J. , Mackenzie, L. , Conway, S. J. , Holmes, A. B. , Bootman, M. D. , … Roderick, H. L. (2003). 2‐Aminoethoxydiphenyl borate (2‐APB) antagonises inositol 1,4,5‐trisphosphate‐induced calcium release, inhibits calcium pumps and has a use‐dependent and slowly reversible action on store‐operated calcium entry channels. Cell Calcium, 34(1), 97–108. 10.1016/S0143-4160(03)00026-5 12767897

[bph14762-bib-0063] Phuong, T. T. T. , Redmon, S. N. , Yarishkin, O. , Winter, J. M. , Li, D. Y. , & Krizaj, D. (2017). Calcium influx through TRPV4 channels modulates the adherens contacts between retinal microvascular endothelial cells. The Journal of Physiology, 595(22), 6869–6885. 10.1113/JP275052 28949006 PMC5685834

[bph14762-bib-0064] Running Deer, J. L. , Hurley, J. B. , & Yarfitz, S. L. (1995). G protein control of Drosophila photoreceptor phospholipase C. The Journal of Biological Chemistry, 270(21), 12623–12628. 10.1074/jbc.270.21.12623 7759511

[bph14762-bib-0065] Rusko, J. , Wang, X. , & Vanbreemen, C. (1995). Regenerative caffeine‐induced responses in native rabbit aortic endothelial‐cells. British Journal of Pharmacology, 115(5), 811–821. 10.1111/j.1476-5381.1995.tb15005.x 8548181 PMC1908507

[bph14762-bib-0066] Saleem, H. , Tovey, S. C. , Molinski, T. F. , & Taylor, C. W. (2014). Interactions of antagonists with subtypes of inositol 1,4,5‐trisphosphate (IP3) receptor. British Journal of Pharmacology, 171(13), 3298–3312. 10.1111/bph.12685 24628114 PMC4080982

[bph14762-bib-0067] Sayed, A. , Schierling, W. , Troidl, K. , Ruding, I. , Nelson, K. , Apfelbeck, H. , … Schmitz‐Rixen, T. (2010). Exercise linked to transient increase in expression and activity of cation channels in newly formed hind‐limb collaterals. European Journal of Vascular and Endovascular Surgery, 40(1), 81–87. 10.1016/j.ejvs.2010.02.014 20304685

[bph14762-bib-0068] Schierling, W. , Troidl, K. , Apfelbeck, H. , Troidl, C. , Kasprzak, P. M. , Schaper, W. , & Schmitz‐Rixen, T. (2011). Cerebral arteriogenesis is enhanced by pharmacological as well as fluid‐shear‐stress activation of the Trpv4 calcium channel. European Journal of Vascular and Endovascular Surgery, 41(5), 589–596. 10.1016/j.ejvs.2010.11.034 21316269

[bph14762-bib-0069] Shen, J. , Tu, L. , Chen, D. , Tan, T. , Wang, Y. , & Wang, S. (2018). TRPV4 channels stimulate Ca^2+^‐induced Ca^2+^ release in mouse neurons and trigger endoplasmic reticulum stress after intracerebral hemorrhage. Brain Research Bulletin, 146, 143–152. 30508606 10.1016/j.brainresbull.2018.11.024

[bph14762-bib-0070] Sonkusare, S. K. , Bonev, A. D. , Ledoux, J. , Liedtke, W. , Kotlikoff, M. I. , Heppner, T. J. , … Nelson, M. T. (2012). Elementary Ca^2+^ signals through endothelial TRPV4 channels regulate vascular function. Science, 336(6081), 597–601. 10.1126/science.1216283 22556255 PMC3715993

[bph14762-bib-0071] Sonkusare, S. K. , Dalsgaard, T. , Bonev, A. D. , Hill‐Eubanks, D. C. , Kotlikoff, M. I. , Scott, J. D. , … Nelson, M. T. (2014). AKAP150‐dependent cooperative TRPV4 channel gating is central to endothelium‐dependent vasodilation and is disrupted in hypertension. Science Signaling, 7(333), ra66 10.1126/scisignal.2005052 25005230 PMC4403000

[bph14762-bib-0072] Strotmann, R. , Schultz, G. , & Plant, T. D. (2003). Ca^2+^‐dependent potentiation of the nonselective cation channel TRPV4 is mediated by a C‐terminal calmodulin binding site. The Journal of Biological Chemistry, 278(29), 26541–26549. 10.1074/jbc.M302590200 12724311

[bph14762-bib-0073] Sullivan, M. N. , Francis, M. , Pitts, N. L. , Taylor, M. S. , & Earley, S. (2012). Optical recording reveals novel properties of GSK1016790A‐induced vanilloid transient receptor potential channel TRPV4 activity in primary human endothelial cells. Molecular Pharmacology, 82(3), 464–472. 10.1124/mol.112.078584 22689561 PMC3422704

[bph14762-bib-0074] Sun, M. Y. , Geyer, M. , & Komarova, Y. A. (2017). IP3 receptor signaling and endothelial barrier function. Cellular and Molecular Life Sciences, 74(22), 4189–4207. 10.1007/s00018-017-2624-8 28803370 PMC5724978

[bph14762-bib-0075] Tallini, Y. N. , Brekke, J. F. , Shui, B. , Doran, R. , Hwang, S. M. , Nakai, J. , … Kotlikoff, M. I. (2007). Propagated endothelial Ca^2+^ waves and arteriolar dilation in vivo: measurements in Cx40BAC GCaMP2 transgenic mice. Circulation Research, 101(12), 1300–1309. 10.1161/CIRCRESAHA.107.149484 17932328

[bph14762-bib-0076] Taylor, M. S. , Bonev, A. D. , Gross, T. P. , Eckman, D. M. , Brayden, J. E. , Bond, C. T. , … Nelson, M. T. (2003). Altered expression of small‐conductance Ca^2+^‐activated K^+^ (SK3) channels modulates arterial tone and blood pressure. Circulation Research, 93(2), 124–131. 10.1161/01.RES.0000081980.63146.69 12805243

[bph14762-bib-0077] Thodeti, C. K. , Matthews, B. , Ravi, A. , Mammoto, A. , Ghosh, K. , Bracha, A. L. , & Ingber, D. E. (2009). TRPV4 channels mediate cyclic strain‐induced endothelial cell reorientation through integrin‐to‐integrin signaling. Circulation Research, 104(9), 1123–1130. 10.1161/CIRCRESAHA.108.192930 19359599 PMC2754067

[bph14762-bib-0078] Thoppil, R. J. , Cappelli, H. C. , Adapala, R. K. , Kanugula, A. K. , Paruchuri, S. , & Thodeti, C. K. (2016). TRPV4 channels regulate tumor angiogenesis via modulation of Rho/Rho kinase pathway. Oncotarget, 7(18), 25849–25861. 10.18632/oncotarget.8405 27029071 PMC5041949

[bph14762-bib-0079] Trebak, M. , Bird, G. S. , McKay, R. R. , & Putney, J. W. Jr. (2002). Comparison of human TRPC3 channels in receptor‐activated and store‐operated modes. Differential sensitivity to channel blockers suggests fundamental differences in channel composition. Journal of Biological Chemistry, 277(24), 21617–21623. 10.1074/jbc.M202549200 11943785

[bph14762-bib-0080] Troidl, C. , Troidl, K. , Schierling, W. , Cai, W. J. , Nef, H. , Mollmann, H. , … Schaper, W. (2009). Trpv4 induces collateral vessel growth during regeneration of the arterial circulation. Journal of Cellular and Molecular Medicine, 13(8B), 2613–2621. 10.1111/j.1582-4934.2008.00579.x 19017361 PMC6512359

[bph14762-bib-0081] Voets, T. , Prenen, J. , Fleig, A. , Vennekens, R. , Watanabe, H. , Hoenderop, J. G. , … Nilius, B. (2001). CaT1 and the calcium release‐activated calcium channel manifest distinct pore properties. The Journal of Biological Chemistry, 276(51), 47767–47770. 10.1074/jbc.C100607200 11687570

[bph14762-bib-0082] Vriens, J. , Watanabe, H. , Janssens, A. , Droogmans, G. , Voets, T. , & Nilius, B. (2004). Cell swelling, heat, and chemical agonists use distinct pathways for the activation of the cation channel TRPV4. Proceedings of the National Academy of Sciences of the United States of America, 101(1), 396–401. 10.1073/pnas.0303329101 14691263 PMC314196

[bph14762-bib-0083] Watanabe, H. , Davis, J. B. , Smart, D. , Jerman, J. C. , Smith, G. D. , Hayes, P. , … Nilius, B. (2002). Activation of TRPV4 channels (hVRL‐2/mTRP12) by phorbol derivatives. The Journal of Biological Chemistry, 277(16), 13569–13577. 10.1074/jbc.M200062200 11827975

[bph14762-bib-0084] Watanabe, H. , Vriens, J. , Janssens, A. , Wondergem, R. , Droogmans, G. , & Nilius, B. (2003). Modulation of TRPV4 gating by intra‐ and extracellular Ca^2+^ . Cell Calcium, 33(5–6), 489–495. 10.1016/S0143-4160(03)00064-2 12765694

[bph14762-bib-0085] Watanabe, H. , Vriens, J. , Prenen, J. , Droogmans, G. , Voets, T. , & Nilius, B. (2003). Anandamide and arachidonic acid use epoxyeicosatrienoic acids to activate TRPV4 channels. Nature, 424(6947), 434–438. 10.1038/nature01807 12879072

[bph14762-bib-0086] Whorton, A. R. , Willis, C. E. , Kent, R. S. , & Young, S. L. (1984). The role of calcium in the regulation of prostacyclin synthesis by porcine aortic endothelial cells. Lipids, 19(1), 17–24. 10.1007/BF02534603 6423923

[bph14762-bib-0087] Willars, G. B. , Nahorski, S. R. , & Challiss, R. A. (1998). Differential regulation of muscarinic acetylcholine receptor‐sensitive polyphosphoinositide pools and consequences for signaling in human neuroblastoma cells. The Journal of Biological Chemistry, 273(9), 5037–5046. 10.1074/jbc.273.9.5037 9478953

[bph14762-bib-0088] Wilson, C. , Lee, M. , & McCarron, J. G. (2016). Acetylcholine released by endothelial cells facilitates flow‐mediated dilatation. The Journal of Physiology, 594, 7267–7307. 10.1113/JP272927 27730645 PMC5157078

[bph14762-bib-0089] Wilson, C. , Lee, M. D. , Heathcote, H. R. , Zhang, X. , Buckley, C. , Girkin, J. M. , … McCarron, J. G. (2019). Mitochondrial ATP production provides long‐range control of endothelial inositol trisphosphate‐evoked calcium signaling. The Journal of Biological Chemistry, 294(3), 737–758. 10.1074/jbc.RA118.005913 30498088 PMC6341391

[bph14762-bib-0090] Wilson, C. , Saunter, C. D. , Girkin, J. M. , & McCarron, J. G. (2015). Pressure‐dependent regulation of Ca^2+^ signaling in the vascular endothelium. The Journal of Physiology, 593, 5231–5253. 10.1113/JP271157 26507455 PMC4704526

[bph14762-bib-0091] Wilson, C. , Saunter, C. D. , Girkin, J. M. , & McCarron, J. G. (2016). Clusters of specialized detector cells provide sensitive and high fidelity receptor signaling in intact endothelium. The FASEB Journal, 30, 2000–2013. 10.1096/fj.201500090 26873937 PMC4836367

[bph14762-bib-0092] Yin, J. , Hoffmann, J. , Kaestle, S. M. , Neye, N. , Wang, L. , Baeurle, J. , … Kuebler, W. M. (2008). Negative‐feedback loop attenuates hydrostatic lung edema via a cGMP‐dependent regulation of transient receptor potential vanilloid 4. Circulation Research, 102(8), 966–974. 10.1161/CIRCRESAHA.107.168724 18323527

[bph14762-bib-0093] Zhang, D. X. , Mendoza, S. A. , Bubolz, A. H. , Mizuno, A. , Ge, Z. D. , Li, R. , … Gutterman, D. D. (2009). Transient receptor potential vanilloid type 4‐deficient mice exhibit impaired endothelium‐dependent relaxation induced by acetylcholine in vitro and in vivo. Hypertension, 53(3), 532–538. 10.1161/HYPERTENSIONAHA.108.127100 19188524 PMC2694062

[bph14762-bib-0094] Zhang, L. , Papadopoulos, P. , & Hamel, E. (2013). Endothelial TRPV4 channels mediate dilation of cerebral arteries: Impairment and recovery in cerebrovascular pathologies related to Alzheimer's disease. British Journal of Pharmacology, 170(3), 661–670. 10.1111/bph.12315 23889563 PMC3792003

