# Supplementary material for: Endothelial TRPV4 channels modulate vascular tone by Ca2+‐induced Ca2+ release at inositol 1,4,5‐trisphosphate receptors
Source: Br J Pharmacol. 2019 Jul 24;176(17):3297–317. doi: 10.1111/bph.14762 (PMC6692577; doi:10.1111/bph.14762)
Supplement: Supplementary file 1 — Data S1: List of IUPHAR Hyperlinks [file BPH-176-3297-s001.docx]

**Supporting Information**

**List of IUPHAR Hyperlinks**

Endothelial TRPV4 channels modulate vascular tone by Ca^2+^-induced Ca^2+^ release at IP_3_ receptors

Helen R. Heathcote^1^, Matthew D. Lee^1^, Xun Zhang^1^, Christopher D. Saunter^2^, Calum Wilson^1^ & John G. McCarron^1^*

^1^Strathclyde Institute of Pharmacy and Biomedical Science, University of Strathclyde,

161 Cathedral Street, Glasgow, G4 0RE, UK

^2^ Centre for Advanced Instrumentation, Biophysical Sciences Institute, Department of Physics, Durham University, South Road, Durham, DH1 3LE, UK

* To whom correspondence should be addressed: John G McCarron, Strathclyde Institute of Pharmacy and Biomedical Science, 161 Cathedral Street, Glasgow, G4 0RE; john.mccarron@strath.ac.uk; Tel +44 (0)141 548 4119

TRPV4: <https://www.guidetopharmacology.org/GRAC/ObjectDisplayForward?objectId=510>

Ryanodine: <https://www.guidetopharmacology.org/GRAC/LigandDisplayForward?ligandId=4303>

RyRs: <https://www.guidetopharmacology.org/GRAC/FamilyDisplayForward?familyId=125>

IP3: <https://www.guidetopharmacology.org/GRAC/LigandDisplayForward?ligandId=4222>

IP_3_Rs: <https://www.guidetopharmacology.org/GRAC/FamilyDisplayForward?familyId=123>

Caffeine: <https://www.guidetopharmacology.org/GRAC/LigandDisplayForward?ligandId=407>

ACh: <https://www.guidetopharmacology.org/GRAC/LigandDisplayForward?ligandId=294>

GSK1016790A: <https://www.guidetopharmacology.org/GRAC/LigandDisplayForward?ligandId=4205>

Phenylephrine: <https://www.guidetopharmacology.org/GRAC/LigandDisplayForward?ligandId=485>

Sodium nitroprusside: <https://www.guidetopharmacology.org/GRAC/LigandDisplayForward?ligandId=9533>

Cyclopiazonic acid: <https://www.guidetopharmacology.org/GRAC/LigandDisplayForward?ligandId=5350>

HC067047 - <https://www.guidetopharmacology.org/GRAC/LigandDisplayForward?ligandId=4213>

U73122 - <https://www.guidetopharmacology.org/GRAC/LigandDisplayForward?ligandId=5283>

2-APB: <https://www.guidetopharmacology.org/GRAC/LigandDisplayForward?ligandId=2433>

Ruthenium red: <https://www.guidetopharmacology.org/GRAC/LigandDisplayForward?ligandId=2432>

Ca2+-ATPase: <https://www.guidetopharmacology.org/GRAC/FamilyDisplayForward?familyId=159>

Phospholipase C: <https://www.guidetopharmacology.org/GRAC/FamilyDisplayForward?familyId=274>

PIP2: <https://www.guidetopharmacology.org/GRAC/LigandDisplayForward?ligandId=2387>
