# Supplementary material for: Endothelial TRPV4 channels modulate vascular tone by Ca2+‐induced Ca2+ release at inositol 1,4,5‐trisphosphate receptors
Source: Br J Pharmacol. 2019 Jul 24;176(17):3297–317. doi: 10.1111/bph.14762 (PMC6692577; doi:10.1111/bph.14762)
Supplement: Supplementary file 2 — Figure S1: Extraction of Ca2+ signals from raw data. A) Ca2+ image displaying a field of 97 native mesenteric artery endothelial cells. The centre of each cell is marked with a red dot. B) F/F0 Ca2+ traces for each of the cells identified in (A). The baseline region (red) was automatically identified for each cell, a trace from a single cell is highlighted in bold. C‐E) To extract signalling metrics, fast and slow Ca2+ signal components were extracted from F/F0 traces. C) Example of Ca2+ signal de‐multiplexing for the cellular trace (#57) highlighted in B. The time‐dependent baseline (slow component, blue) of the F/F0 signal (black) was calculated using an asymmetric least‐squares (ALS) function, and modelled by a sigmoid function (green). The fast component of the signal (red) was isolated by dividing the F/F0 signal by the ALS signal. D) Sigmoid function, used to extract the rise time of the slow signal component, and fast signal component (shown in C) shown on an expanded time scale. Peaks were identified in the fast signal using an automated peak detection algorithm. E) Original F/F0, de‐multiplexed signal components, and sigmoid fits for each of the signals shown in B. Each trace is coloured according to the magnitude of the ALS signal, mean signal is overlaid in black. Figure S2: ACh and GSK dilate PE‐constricted mesenteric arteries. A) Mean PE‐contracted vessel diameter (% of resting, left) and corresponding concentration of PE (right), before (+ endothelium) and after (‐ endothelium) mechanical removal of the endothelium (n = 10). B) Mean PE‐contracted vessel diameter (% of resting, left) and corresponding concentration of PE (right), in the absence (‐) and presence (+) of cyclopiazonic acid (CPA; 6 μM; n = 5). C) Summary data showing the effect of ACh (blue) and GSK (red) on the diameter of PE‐constricted arteries. Statistical analyses were performed using paired Student's t‐test (A), unpaired Student's t‐test (B) and repeated measures one‐way ANOVA with mu [file BPH-176-3297-s002.pdf]

SUPPLEMENTARY INFORMATION

Endothelial TRPV4 channels modulate vascular tone by Ca^{2+}-induced Ca^{2+} release at IP_3 receptors

Helen R. Heathcote[1], Matthew D. Lee[1], Xun Zhang[1], Christopher D. Saunter[2], Calum Wilson[1] & John G. McCarron[1]*

[1]Strathclyde Institute of Pharmacy and Biomedical Science, University of Strathclyde, 161 Cathedral Street, Glasgow, G4 0RE, UK

[2] Centre for Advanced Instrumentation, Biophysical Sciences Institute, Department of Physics, Durham University, South Road, Durham, DH1 3LE, UK

* To whom correspondence should be addressed: John G McCarron, Strathclyde Institute of Pharmacy and Biomedical Science, 161 Cathedral Street, Glasgow, G4 0RE; john.mccarron@strath.ac.uk; Tel +44 (0)141 548 4119

Supplementary Movie Legends

Supplementary Movie S1: Endothelial Ca^{2+} signalling evoked by GSK. The grey channel shows basal Ca^{2+} levels, whilst the red channel shows Ca^{2+} activity (red, determined by sequential subtraction) evoked by the TRPV4 agonist, GSK1016790A. Data acquired at 10 Hz. Scale bar = 20 µm.

Supplementary Movie S2: GSK-evoked multicellular Ca^{2+} waves. Pseudo-coloured active Ca^{2+} wave fronts (determined by sequential subtraction) showing a GSK-evoked (20 nM) Ca^{2+} wave that propagates across four endothelial cells. Cell outlines are overlaid in white. Data acquired at 20 Hz. Scale bar = 20 µm.

Supplementary Movie S3: Ca^{2+} waves stop upon washout of GSK. The grey channel shows basal Ca^{2+} levels, whilst the red channel shows Ca^{2+} activity (red, determined by sequential subtraction). GSK (20 nM) present from start of video and washed out when indicated. Data acquired at 10 Hz. Scale bar = 20 µm.

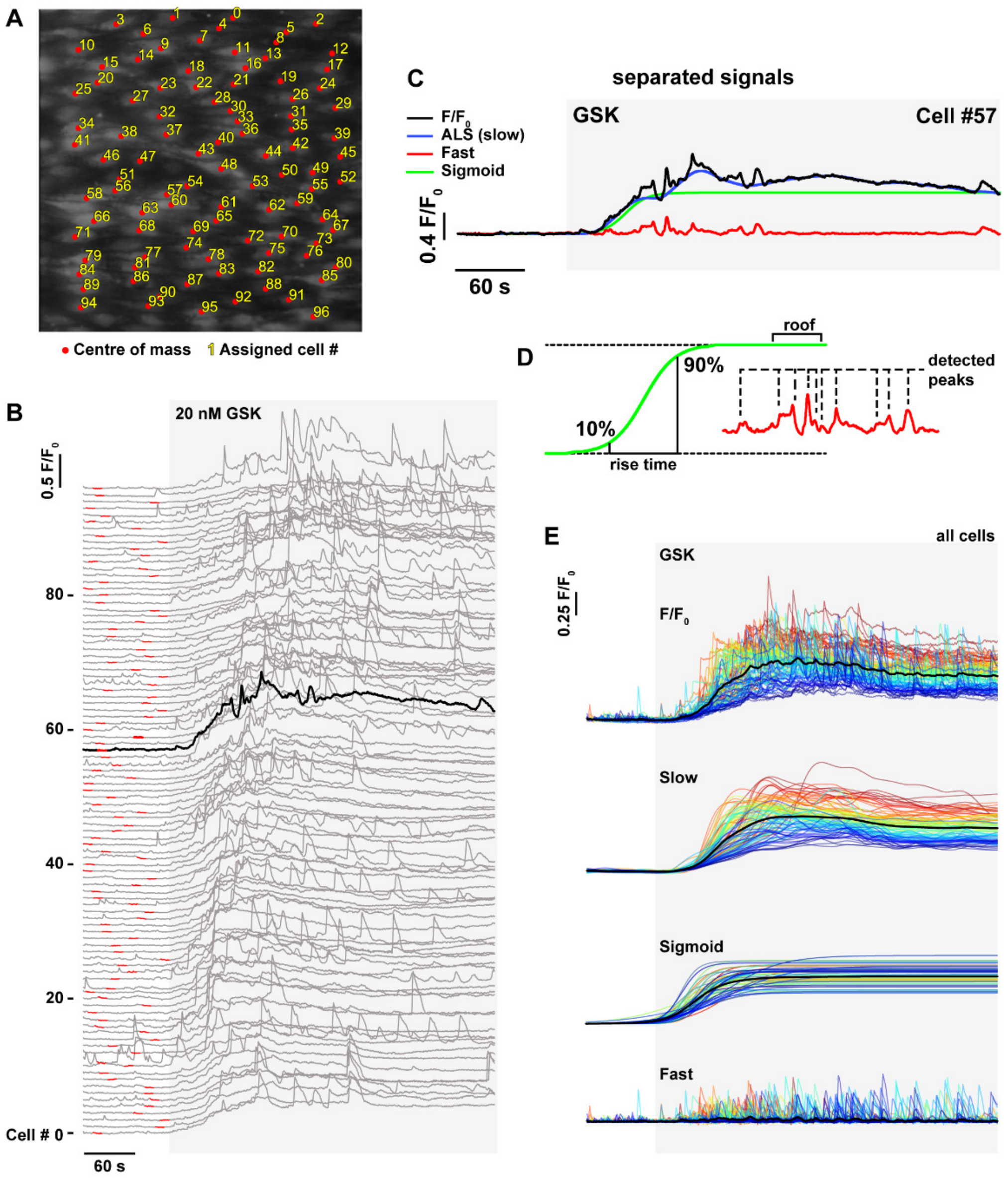

Supplementary Figure S1: Extraction of Ca^{2+} signals from raw data. A) Ca^{2+} image displaying a field of 97 native mesenteric artery endothelial cells. The centre of each cell is marked with a red dot. B) F/F_0 Ca^{2+} traces for each of the cells identified in (A). The baseline region (red) was automatically identified for each cell, a trace from a single cell is highlighted in bold. C-E) To extract signalling metrics, fast and slow Ca^{2+} signal components were extracted from F/F_0 traces. C) Example of Ca^{2+} signal de-multiplexing for the cellular trace (#57) highlighted in B. The time-dependent baseline (slow component, blue) of the F/F_0 signal (black) was calculated using an asymmetric least-squares (ALS) function, and modelled by a sigmoid function (green). The fast component of the signal (red) was isolated by dividing the F/F_0 signal by the ALS signal. D) Sigmoid function, used to extract the rise time of the slow signal component, and fast signal component (shown in C) shown on an expanded time scale. Peaks were identified in the fast signal using an automated peak detection algorithm. E) Original F/F_0, de-multiplexed signal components, and sigmoid fits for each of the signals shown in B. Each trace is coloured according to the magnitude of the ALS signal, mean signal is overlaid in black.

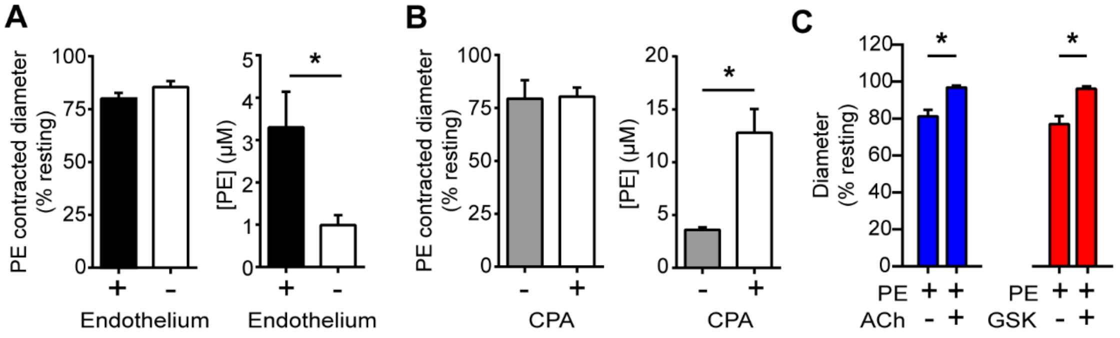

Supplementary Figure S2: ACh and GSK dilate PE-constricted mesenteric arteries. A) Mean PE-contracted vessel diameter (% of resting, left) and corresponding concentration of PE (right), before (+ endothelium) and after (- endothelium) mechanical removal of the endothelium (n = 10). B) Mean PE-contracted vessel diameter (% of resting, left) and corresponding concentration of PE (right), in the absence (-) and presence (+) of cyclopiazonic acid **(**CPA; 6 µM; n = 5). C) Summary data showing the effect of ACh (blue) and GSK (red) on the diameter of PE-constricted arteries. Statistical analyses were performed using paired Student's *t*-test (A), unpaired Student's *t*-test (B) and repeated measures one-way ANOVA with multiple comparisons (C). * = $p < 0.05$.

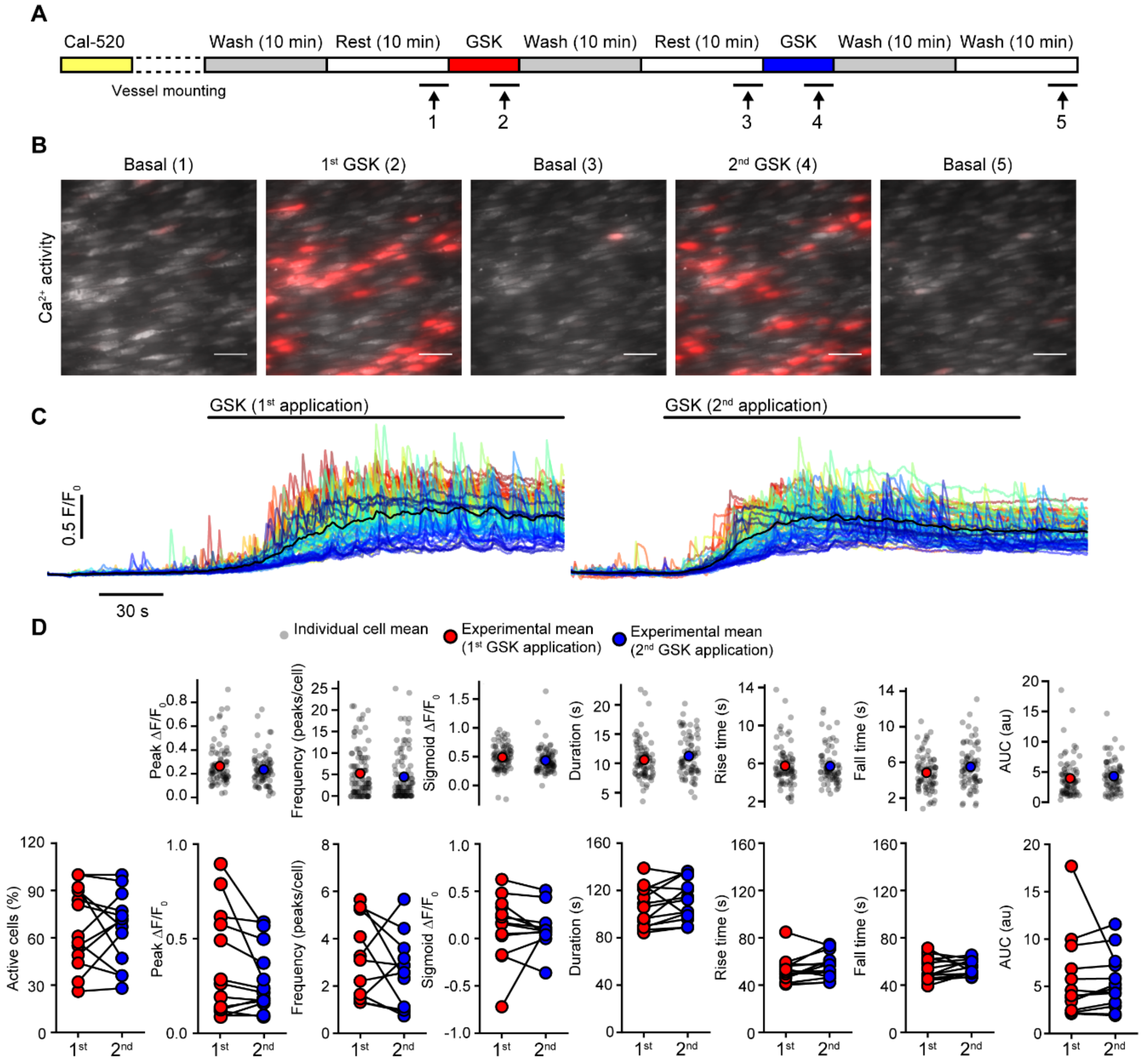

Supplementary Figure S3: Repeat application of GSK elicits reproducible Ca^{2+} signals. A) Schematic of the experimental protocol. Mesenteric artery endothelial cells were subjected to perfusion with GSK1016790A (GSK; 20 nM) prior to washing, and a second application of GSK (20 nM). B) Composite images showing basal Cal-520/AM fluorescence (grey) with Ca^{2+} activity overlaid (red). Ca^{2+} activity was derived from the 120 sec periods indicated by the numbered arrows in A. Scale bars = 50 µm. C) GSK-evoked Ca^{2+} signals for the 1st (left) and 2nd (right) application of GSK. D) GSK-evoked Ca^{2+} signalling parameters. Upper panels show the mean response for individual cells (grey dots) for a single experiment. The experimental mean is overlaid in red or blue. Lower panel show paired summary data (grand mean) for all experimental replicates. Parameters extracted include: the percentage of cells activated, the mean $\Delta F/F_0$ of Ca^{2+} peaks, the number of peaks detected per cell (peaks/cell), the amplitude of the fitted sigmoid, as well as the duration, rise time, fall time, area under the curve (AUC) of all Ca^{2+} signal peaks. * $p < 0.05$ (n=13) using paired Student's *t*-test.

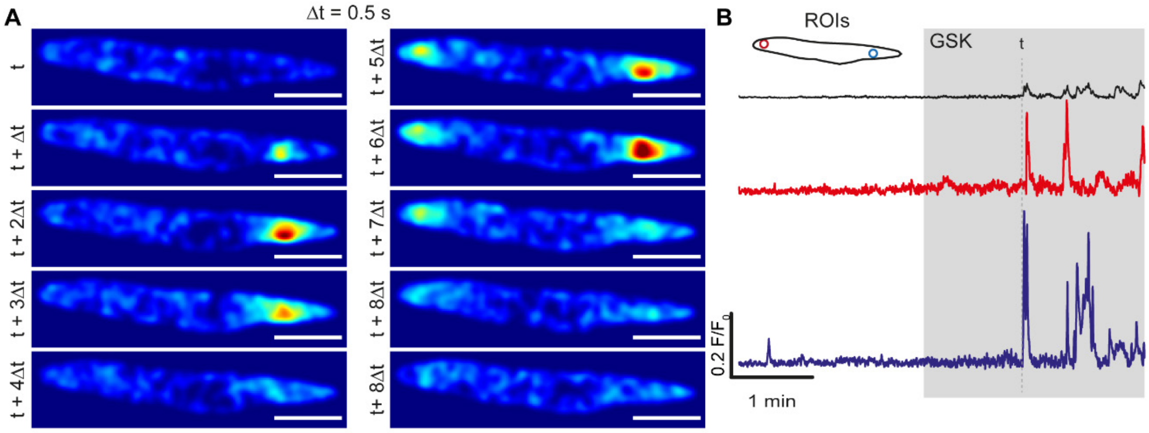

Supplementary Figure S4: TRPV4-mediated local Ca^{2+} activity. A) Local Ca^{2+} events elicited by GSK101679A (GSK; 20 nM). Panels show a $\Delta F/F_0$ time series of a single endothelial cell. Ca^{2+} events initiate at, but remain localized within, either end of the cell. Scale bars = 20 µm. B) Ca^{2+} traces from the entire cell (red) or from circular ROIs (~2.5 µm diameter) positioned at each of the Ca^{2+} event initiation sites. Local Ca^{2+} events are inconsistently reflected in global (whole-cell) Ca^{2+} traces.

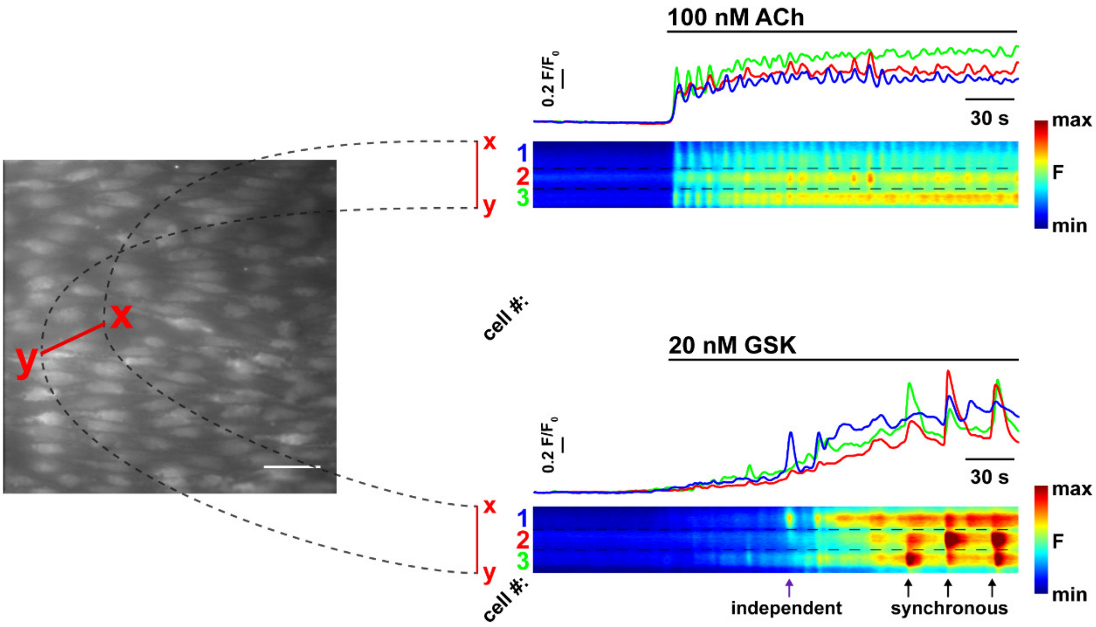

Supplementary Figure S5: Acetylcholine and GSK101679A stimulate different patterns of signalling across multiple cells. Left; Image showing the basal Cal-520/AM fluorescence (grey) in a field of mesenteric artery endothelial cells. Scale bar = 50 µm. A transect (x-y) was drawn through three adjoining cells (1-3). Right) Ca^{2+} signals (F/F_0) and kymographs illustrating Cal-520/AM fluorescence (F) along the transect (x-y; vertical axis) for cells 1-3, when stimulated with acetylcholine (ACh; 100 nM; upper right) and then, after washout and recovery, GSK101679A (GSK; 20 nM; lower right). Traces are coloured according to the assigned cell number and kymographs are coloured on a scale where dark blue = min F and dark red = max F.

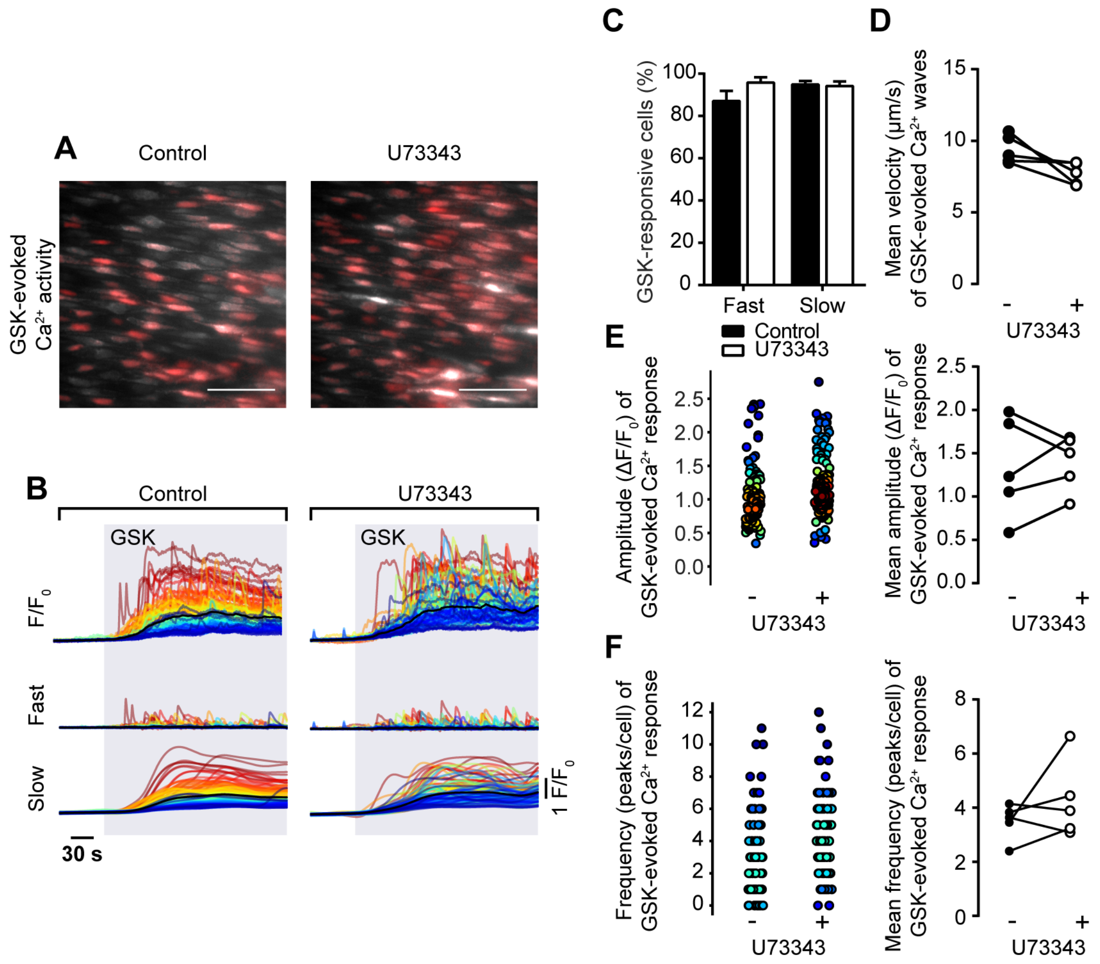

Supplementary Figure S6: U73343, the inactive analogue of U73122, did not alter GSK induced Ca^{2+} signals. A) Composite images showing GSK1016790A (GSK)-evoked Ca^{2+} activity in the absence (left) and presence (right) of U73343 (2 µM; 10 mins), the inactive analogue of the PLC inhibitor U73122 (Figure 6). Images show basal Cal-520/AM fluorescence (grey) with Ca^{2+} activity overlaid (red). Scale bars = 50 µm. B) GSK-evoked (20 nM; grey box) Ca^{2+} signals (F/F_0; top) were decomposed into propagating Ca^{2+} waves (fast; middle) and slow global Ca^{2+} rise (slow; bottom) components. C) Summary data illustrating the percentage of cells displaying propagating Ca^{2+} waves and slow global Ca^{2+} rises. D-F) Paired summary data showing the effect of U73343 on Ca^{2+} wave propagation velocity (D), peak amplitude (ΔF/F0, E) and the number of peaks per cell (F). The left plots in E-F are scatter plots showing the mean Ca^{2+} event amplitude, or oscillation frequency, within each cell visualised in the experiment shown in panels A-B. Individual data points are coloured (from blue, low to red, high) according to the density (i.e. occurrence) of particular values. * $p < 0.05$ using paired Student's t-test ($n = 5$).

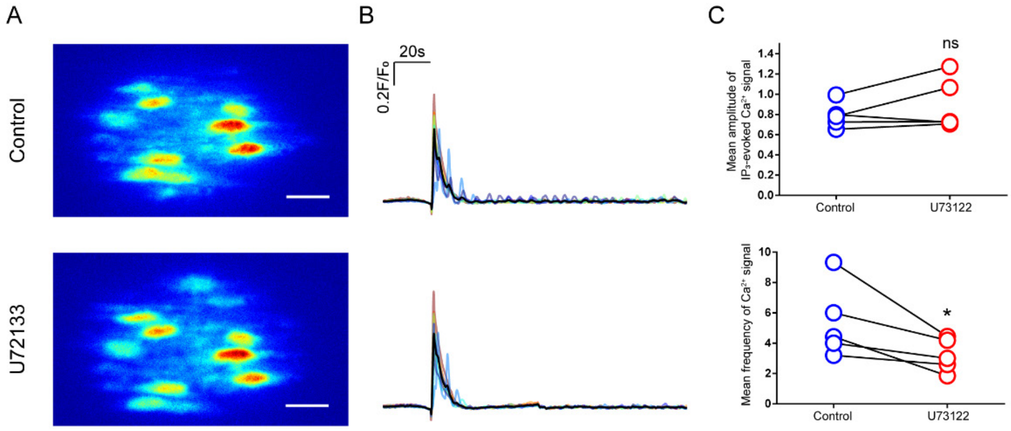

Supplementary Figure S7: U73122 does not alter IP_3-mediated Ca^{2+} signals. A) Composite image (max F/F_0 intensity projection) illustrating the Ca^{2+} response to photolysis of caged-IP_3 in the absence (upper) and presence (lower) of U73122 (2 µM, 10 mins). B) Whole-cell Ca2+ traces extracted from the data shown in A. C) Summary data showing the effect of U73122 on the peak amplitude and number of cells that responded to photorelease of caged IP_3. Each data point indicates the mean from a single field of endothelial cells (one animal). *, $p < 0.05$; NS, no statistically significant difference detected (i.e. $p > 0.05$) using paired t test. Scale bars = 10 µm.

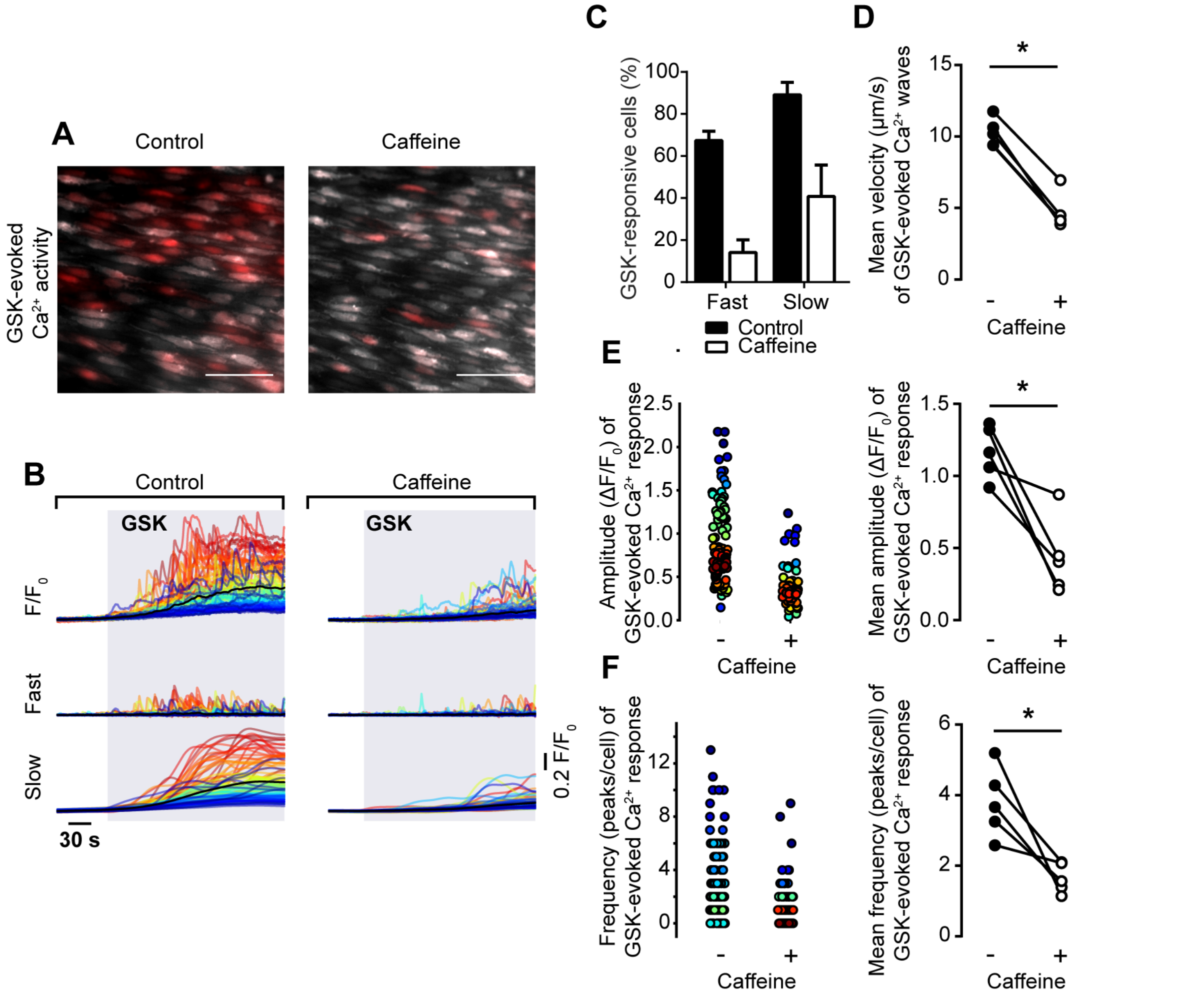

Supplementary Figure S8: Caffeine inhibits propagating Ca^{2+} waves induced by TRPV4 activation. A) Composite images showing GSK1016790A (GSK)-evoked Ca^{2+} activity in native mesenteric artery endothelial cells in the absence (left) and presence of caffeine (10 mM; right). Images show basal Cal-520/AM fluorescence (grey) with Ca^{2+} activity overlaid (red). Scale bars = 50 µm. B) Raw F/F_0 (top), propagating Ca^{2+} waves (fast; middle), and slow Ca^{2+} rises (slow; bottom) GSK-evoked (20 nM; grey box) Ca^{2+} signal components in the absence (left) and presence (right) of caffeine. C) Summary data illustrating the percentage of cells exhibiting fast and slow signal components. D-F) Paired summary data showing the effect of IP_3R inhibition on Ca^{2+} wave propagation velocity (D), peak amplitude ($\Delta F/F_0$, E) and the number of peaks per cell (F). The left plots in E-F are scatter plots showing the mean Ca^{2+} event amplitude, or oscillation frequency, within each cell visualised in the experiment shown in panels A-B. Individual data points are coloured (from blue, low to red, high) according to the density (i.e. occurrence) of particular values. * $p < 0.05$ using paired Student's t-test ($n = 5$).

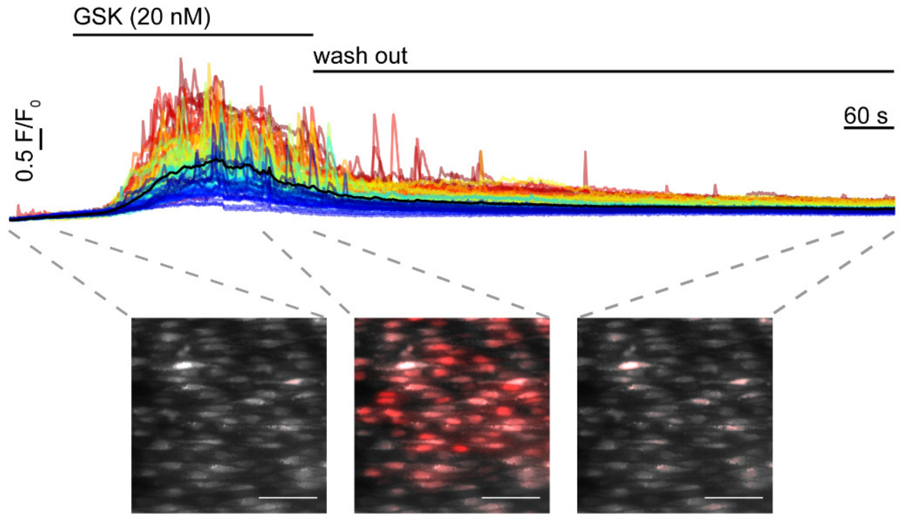

Supplementary Figure S9: GSK-evoked Ca^{2+} signalling returns towards basal levels during a washout period. Ca^{2+} signals (F/F_0; top) extracted from mesenteric artery endothelial cells stimulated with GSK1016790A (GSK; 20 nM). When GSK was washed out, signals returned to resting levels. Composite images (bottom) showing basal Cal-520/AM fluorescence (grey) with Ca^{2+} activity overlaid (red). Ca^{2+} activity was derived from the 60 sec periods indicated by the dashed lines. Scale bars = 50 µm.

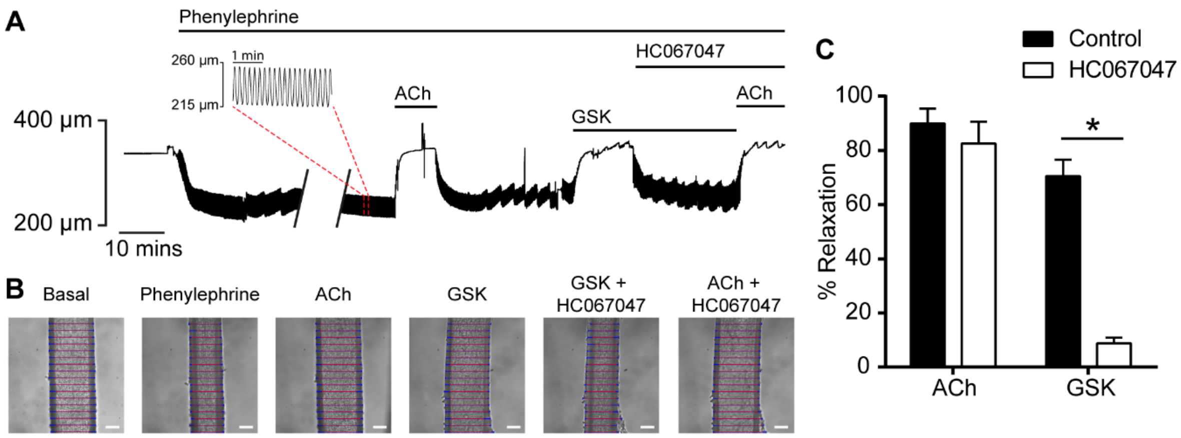

Supplementary Figure S10: Acetylcholine (ACh) evoked relaxation is independent of TRPV4 in rat mesenteric arteries. (A) Representative diameter trace of a second order mesenteric artery (60 mmHg; luminal flow rate ~100 µl/min) in response to ACh or GSK. Upon exposure to PE (~500 nM; duration indicated by the bar above the trace), vessels contracted and underwent vasomotion. Upon subsequent exposure to ACh (100 nM), arteries relaxed back towards resting diameter. On wash-out of ACh, arteries contracted again. GSK (20 nM) also relaxed arteries back towards resting diameter. Dilation to GSK was reversed by the TRPV4 antagonist, HC067047 (10 µM). However, HC067047 did not prevent dilation to ACh. (B) Example images from the artery in A showing the artery prior to PE exposure (basal), after PE, after ACh, after GSK, after GSK and HC067047, and after ACh with HC067047. The red horizontal lines are measurement scan lines created by Vasotracker (see Methods). (C) Summary data showing the percentage relaxation to ACh and GSK1016790A (GSK) in the absence (black bar) and present (open bar) of HC067047 (n=5; * $p<0.05$).
